# The global, regional, and national burden of stomach cancer among adolescents and young adults in 204 countries and territories, 1990–2019: A population-based study

**DOI:** 10.3389/fpubh.2023.1079248

**Published:** 2023-02-24

**Authors:** Ziqiang Zhang, Jun Wang, Ning Song, Liubin Shi, Jianjun Du

**Affiliations:** Department of General Surgery, Huashan Hospital, Fudan University, Shanghai, China

**Keywords:** disease burden, stomach cancer, incidence, mortality, adolescents, young adults

## Abstract

**Background:**

Stomach cancer is a significant health problem in many countries. But healthcare needs of adolescents and young adults (AYAs) stomach cancer patients have been historically neglected. An accurate appraisal of the burden of AYA stomach cancer is crucial to formulating effective preventive strategies. In this study, we report the most recent estimates of AYA stomach cancer burden concerning socio-demographic index (SDI) in 204 countries and territories between 1990 and 2019.

**Methods:**

Estimates from the Global Burden of Disease study 2019 were used to analyze incidence, mortality, and disability-adjusted life years (DALYs) due to AYA stomach cancer at global, regional, and national levels. Association between AYA stomach cancer burden and SDI were investigated. All estimates are reported as absolute numbers and age-standardized rates, which were standardized to the GBD world population and reported per 100,000 population.

**Results:**

In 2019, there were 49,000 incident cases, 27,895 deaths, and 1.57 million DALYs due to AYA stomach cancer globally. The highest age-standardized incidence rate occurred in East Asia [2.42 (women) and 4.71 (men) per 100,000 person-years] and high-income Asia Pacific [3.16 (women) and 2.61 (men) per 100,000 person-years]. Age-standardized death [1.53 (women) and 2.65 (men) per 100,000 person-years] and DALY [150.96 (women) and 87.13 (men) per 100,000 person-years] rates were highest in Oceania. Compared with 1990, in 2019 more than 1,075 more incident cases of AYA stomach cancer were estimated with a decrease of 7,784 deaths. Despite the increase in absolute number of incident cases, the worldwide age-standardized rates of AYA stomach cancer (incidence, deaths, and DALYs) have declined since 1990. The drop in the disease burden was associated with an improved SDI. Globally, 24.41% of the age-standardized DALYs were attributable to a high-sodium diet in both sexes combined, and 0.57% of the age-standardized DALYs were attributable to smoking in men.

**Conclusion:**

The global burden of AYA stomach cancer is substantial, especially in developing regions. Capacity-building activities for AYA stomach cancer will benefit the younger generation and population health worldwide.

## 1. Introduction

Stomach cancer accounts for a large proportion of the cancer burden (1) and is the most common cancer worldwide (2). The incidence and mortality rates of stomach cancer have declined over the last century (3). Tumors near or away from the gastroesophageal junction (cardia) are classified as cardia and non-cardia tumors, respectively (1). In most populations, the incidence and mortality rates of stomach cancer have decreased due to a decline in non-cardia stomach cancer rates, a trend associated with *Helicobacter pylori* infections (4, 5). It is known that *H. pylori* can be regarded as a carcinogen in non-cardia stomach cancer. Most adults are infected by *H. pylori* once in their lives (6). A decrease in infection rate can be attributed to improved socioeconomic status, hygiene practices, and widespread antibiotic use (7).

The prognosis of stomach cancer remains poor, even though the survival rate has improved over the past decades (8). The 5-year survival rate is ~20%. However, the survival rates were 65% in Japan (9) and 71.5% in South Korea (10), where population screening has improved the early-stage tumor detection rate (11). Nevertheless, some researchers imply that stomach cancer rates may be increasing among younger population in the United States, which might reverse the overall decrease in incidence (12, 13). South Korea has the highest rate of young patients with stomach cancer, with 15% diagnosed before the age of 45 years (14). There are striking differences in the genetic, proteomic, and clinical characteristics between early-onset stomach cancer (under 40 years old) and traditional late-onset stomach cancer (over 40 years old) (15–17). Early-onset stomach cancer displays a more diffuse histology, are highly metastatic, behave aggressively, exhibit molecular heterogeneity, and have poorer prognoses (17, 18).

Cancer in children and older adults has been a focus of research but recent studies have estimated a rising global burden of stomach cancer in adolescent and young adult (AYA) patients (19, 20). The treatment for children would bring them decades of quality life and the incidence of most epithelial cancers increases with age. Thus, to some degree, cancers that occur during young adulthood, as a transition between pediatric and adult oncology, have received less attention from researchers. These types of cancers represent a distinct spectrum of illnesses occurring in younger people who have a large part of their expected lifespans remaining, contribute substantially to the economy and care for their families (21).

Consequently, studies on the specific issues unique to AYA patients with stomach cancer can improve the stomach cancer-related outcomes. In this dedicated analysis of the most recent data from the Global Burden of Diseases, Injuries, and Risk Factors Study (GBD) 2019, we assessed the burden of AYA stomach cancer, described the AYA cancers tendency from 1990 to 2019 and evaluated the diversities caused by levels of human development and geographical differences, which can promote international and local interventions to reduce the disease burden and, perhaps, curb the rising incident cases numbers.

## 2. Methods

### 2.1. Overview

The global, regional, and national AYA stomach cancer cases were determined based on the GBD 2019. According to the International Classification of Diseases 10th edition (ICD-10), GBD 2019 divided cancers into 29 groups (22). Stomach cancer was coded as C16.0–C16.9 (malignant neoplasm of the stomach), Z12.0, and Z85.02-Z85.028 but did not include gastroesophageal junction tumors (23). The present analysis used the data on cancer incidence, mortality, and disability-adjusted life years (DALYs) estimates to assess the cancer burden. A query tool provided by the Global Health Data Exchange was used to retrieve the data for this study.

### 2.2. Estimation of cancer burden

A series of capstone publications summarized the methodology of GBD 2019 in detail (24–27). Data on mortality were obtained from vital registrations and verbal autopsies. The estimated mortality data were multiplied by high-quality cancer registered incidence cases by the independently modeled mortality-to-incidence ratio (MIR). A linear-step mixed-effects model was used to estimate the MIRs, which were smoothed and adjusted using spatiotemporal Gaussian process modeling (22). The modeled mortality estimates were derived from a cause of death ensemble model, including both the observed and estimated death data (24). The incidence estimate was calculated by dividing the modeled mortality estimate by the MIR. Disability-adjusted life-years (DALYs) were obtained by adding years lived with disability (YLDs) and years of life lost (YLLs).

### 2.3. Socio-demographic index

The level of development was measured by using sociodemographic index (SDI), which is strongly associated with health outcomes. This measure is a combination of the total fertility rate of people who are <25 years old, the mean education achieved by people who are more than 15 years old, and the lag distributed income for each person. According to the GBD 2019 studies, SDI and values are available for each country by year (24).

### 2.4. Data analysis

As previously defined by AYA oncology, the study age group was 15–39 years (28). Based on the GBD 2019 study, data on stomach cancer burden among AYA patients were extracted at global, regional, and national levels for both sexes from 1990 to 2019. Cancer data were structured according to age groups (15–19, 20–24, 25–29, 30–34, and 35–39 years-old). The total number of incidence cases, deaths, and DALYs in all related aged groups (15–19 years to 35–39 years) were calculated. The truncated age standardized rates were computed using age-specific rate for age-groups, weighed using the GBD 2019 world standard population (24), according to methods proposed by Boyle and Parkin (29).

The outcomes were presented at the global, regional, and national levels for AYA stomach cancer. The SDI was analyzed using the LOWESS regression method to determine its association with age-standardized incidence, death, and DALY rates. With regard to the cancer burden, uncertainty exists owing to the diversity in availability and quality. Based on the 2.5th and 97.5th percentiles of the allocation of 1,000 draws within the Bayesian modeling, 95% uncertainty intervals (95% UI) were calculated in conjunction with evaluations of cancer burden.

The Joinpoint regression program, version 4.9.1.0 (Statistical Research and Applications Branch, National Cancer Institute, Bethesda, MD), was used to calculate the annual percent changes (APCs) and 95% confidence intervals (CIs) in age-standardized rates as described (30).

## 3. Results

### 3.1. Global incidence, deaths, and DALYs due to AYA stomach cancer

In 2019, stomach cancer was the fifth leading cause of cancer-related death in adolescents and young adults worldwide (31). In the same year, more than 49,000 (95% UI: 45,008–53,078) AYA stomach cancer incident cases occurred globally, and 27,895 individuals (25,711–30,240) died (Table 1). The age-standardized incidence rate of AYA stomach cancer was 1.62 per 100,000 population. Meanwhile, the age-standardized mortality rate was 0.92 per 100,000 population. In 2019, the number of DALYs related to AYA stomach cancer worldwide was 1.57 million (1.45–1.70). Compared to that of women, men with AYA stomach cancer had higher age-standardized incidence and death rates (1.82 vs. 1.41 and 0.98 vs. 0.86 per 100,000 population, respectively).

**Table 1:** Incident cases, deaths, and DALYs of AYA stomach cancer in 2019, and percentage change of age-standardized rates by sex and GBD region.

	**Incidence**			**Deaths**			**DALYs**		
	**Number of incident cases**	**Age-standardized incidence rate**	**Percentage change in rates, 1990–2019**	**Number of deaths**	**Age-standardized death rate**	**Percentage change in rates, 1990–2019**	**Number of DALYs**	**Age-standardized DALYs rate**	**Percentage change in rates, 1990–2019**
Global	49,008 (45,008–53,078)	1.62	−30.90	27,895 (25,711–30,240)	0.92	−46.77	1573897 (1,448,736–1,703,548)	51.96	−46.43
Male	27,882 (24,956–31,033)	1.82	−22.74	14,972 (13,643–16,520)	0.98	−42.98	8,41,920 (766,656–927,599)	55.08	−42.56
Female	21,126 (18,841–23,541)	1.41	−39.30	12,924 (11,550–14,339)	0.86	−50.55	7,31,976 (653,421–814,243)	48.80	−50.24
East Asia	21,314 (18,107–24,881)	3.59	1.09	8,749 (7,519–10,098)	1.47	−46.3	4,92,053 (424,301–567,121)	82.86	−45.58
Southeast Asia	2,242 (1,924–2,634)	0.81	−44.8	1,643 (1,416–1,895)	0.59	−50.69	92,752 (80,019–106,784)	33.37	−50.52
Oceania	134 (98–178)	2.60	−0.01	107 (79–143)	2.09	−0.49	6,123 (4,518–8,137)	119.08	−0.10
Central Asia	744 (664–838)	1.90	−50.12	594 (529–672)	1.52	−51.67	33,384 (29,749–37,657)	85.29	−51.65
Central Europe	364 (315–418)	0.85	−47.21	249 (216–284)	0.58	−54.93	13,702 (11,901–15,633)	32.10	−54.84
Eastern Europe	2,037 (1,794–2,303)	2.37	−35.89	1,136 (1,002–1,279)	1.31	−50.16	62,836 (55,488–70,647)	73.15	−50
High-income Asia Pacific	1,767 (1,550–2,024)	2.88	−62.27	518 (480–559)	0.84	−77.25	29,127 (27,127–31,388)	47.58	−77.11
Australasia	74 (57–96)	0.69	−22.00	28 (24–33)	0.26	−43.56	1,573 (1,343–1,852)	14.65	−43.63
Western Europe	1,178 (1,010–1,367)	0.79	−38.38	496 (461–533)	0.33	−56.76	27,597 (25,590–29,611)	18.53	−56.74
Southern Latin America	301 (227–388)	1.14	−25.69	191 (165–217)	0.73	−37.97	10,714 (9,322–12,178)	40.74	−37.67
High-income North America	915 (791–1,064)	0.71	−0.50	376 (353–405)	0.29	−19.94	21,101 (19,825–22,685)	16.28	−19.65
Caribbean	197 (153–242)	1.09	−14.56	152 (117–187)	0.84	−17.49	8,589 (6,615–10,537)	40.24	−17.29
Andean Latin America	633 (486–799)	2.51	−24.16	457 (351–581)	1.81	−32.82	26,011 (20,099–32,995)	102.90	−32.82
Central Latin America	1,938 (1,604–2,315)	1.94	−2.87	1,227 (1,024–1,457)	1.23	−20.74	69,591 (58,199–82,570)	69.75	−20.6
Tropical Latin America	1,061 (998–1,130)	1.12	−30.51	773 (726–819)	0.81	−37.81	43,275 (40,729–45,836)	45.55	−37.45
North Africa and Middle East	2,770 (2,341–3,277)	1.03	−36.67	1,996 (1,658–2,398)	0.74	−44.35	112,629 (93,260–134,788)	42.04	−44.05
South Asia	8,655 (7,508–9,913)	1.17	−26.68	6,981 (6,075–8,025)	0.95	−28.43	397,614 (346,382–456,558)	53.71	−28.83
Central sub-Saharan Africa	342 (249.5–439)	0.77	−41.02	279 (204–364)	0.64	−40.46	15,836 (11,579–20,587)	35.76	−40.63
Eastern sub-Saharan Africa	1,109 (908–1,387)	0.80	−44.11	923 (748–1,155)	0.67	−43.05	52,086 (42,259–65,025)	37.52	−43.13
Southern sub-Saharan Africa	250 (199–310)	0.74	−46.28	203 (162–251)	0.60	−47.78	11,329 (9,036–14,000)	33.36	−47.57
Western sub-Saharan Africa	982 (791–1,197)	0.65	−28.09	815 (657–1,001)	0.55	−27.88	45,975 (37,045–56,553)	30.57	−27.95

The absolute numbers and age-specific rates of incidence, deaths, and DALYs for AYA stomach cancers in 2019, along with the associated 95% UIs, are presented by age group in Supplementary Tables S1–S5. From 1990 to 2019, the incidence rate per 100,000 annually decreased in all age groups [ages 15–19 years (from 0.36 to 0.21, APC = −1.84%, *p* < 0.001); ages 20–24 years (from 0.81 to 0.56, APC = −1.26%, *p* < 0.001); ages 25–29 years (from 1.53 to 1.11, APC = −1.02%, *p* < 0.001); ages 30–34 years (from 3.33 to 2.54, APC = −0.91%, *p* < 0.001); and ages 35–39 years (from 6.37 to 4.12, APC = −1.54%, *p* < 0.001)]. However, the absolute incidence amount still increased, especially in East Asia, South Aisa, and high-income North America (among 25–29, 30–34, and 35–39 years, especially in males; Supplementary Figures S1–S5). Recently, the overall death and DALY rates associated with AYA stomach cancer showed a declining trend. However, the number of deaths and DALYs increased among ages (20–24, 25–29, 30–34, and 35–39 years; both male and female) in South Asia from 1990 to 2019 (Supplementary Figures S1–S5).

**Figure 1:**
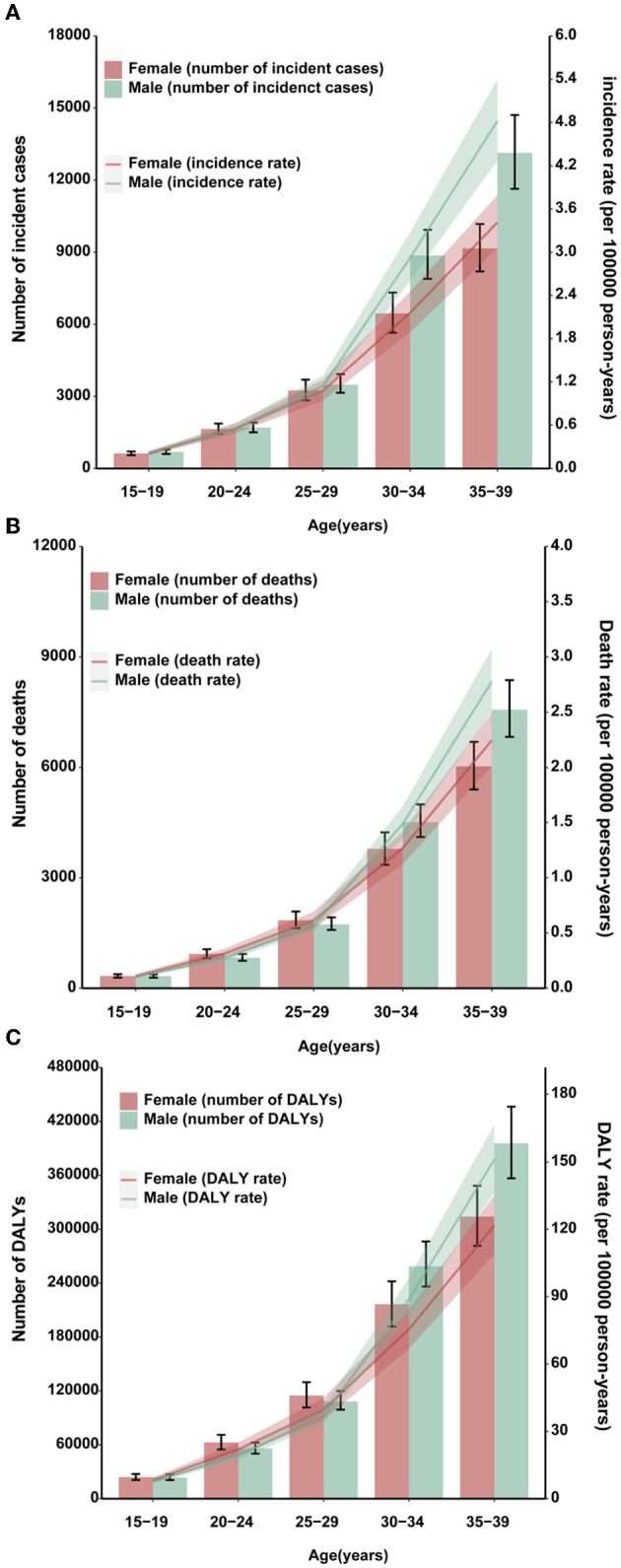
Burden of AYA stomach cancer in 2019. Global age-specific counts and rates of incident cases **(A)**, deaths **(B)**, and DALYs **(C)** of AYA stomach cancers per 100,000 population by sex, 2019.

In both sexes, the absolute numbers of stomach cancer incident cases and deaths, along with the age-standardized rates, increased commensurately as the age increased (Figures 1A–C). Stomach cancer was the major contributor to the AYA cancer burden among 30- to 39-year age groups (31). Consistent with the results of the previous studies, we found an accelerated increase in stomach cancer burden after age 30 for AYAs across all three measures.

**Figure 2:**
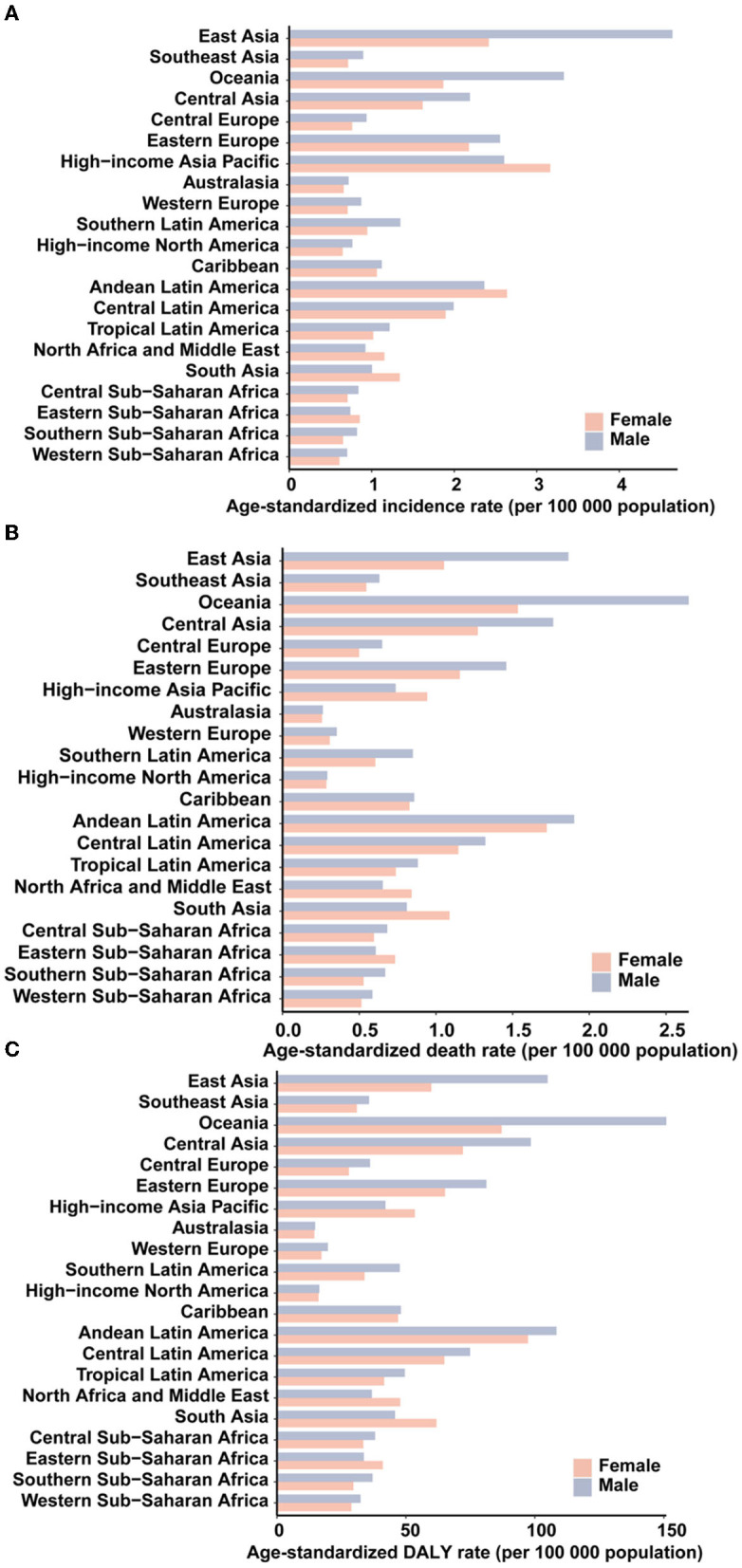
The age-standardized incidence **(A)**, death **(B)**, and DALY **(C)** rates of stomach cancer in 2019 for 21 GBD regions, by sex.

All regions experienced male-female incidence, death and DALY gap but the age-standardized incidence rates of AYA stomach cancer for men were lower than those in women in the high-income Asia Pacific, North Africa, and Middle East, South Asia, and Eastern sub-Saharan Africa (Figures 2A–C). The incidence, deaths, and DALYs for specific countries and territories are presented in Supplementary Table S6.

### 3.2. Regional variations in AYA stomach cancer

**Figure 3:**
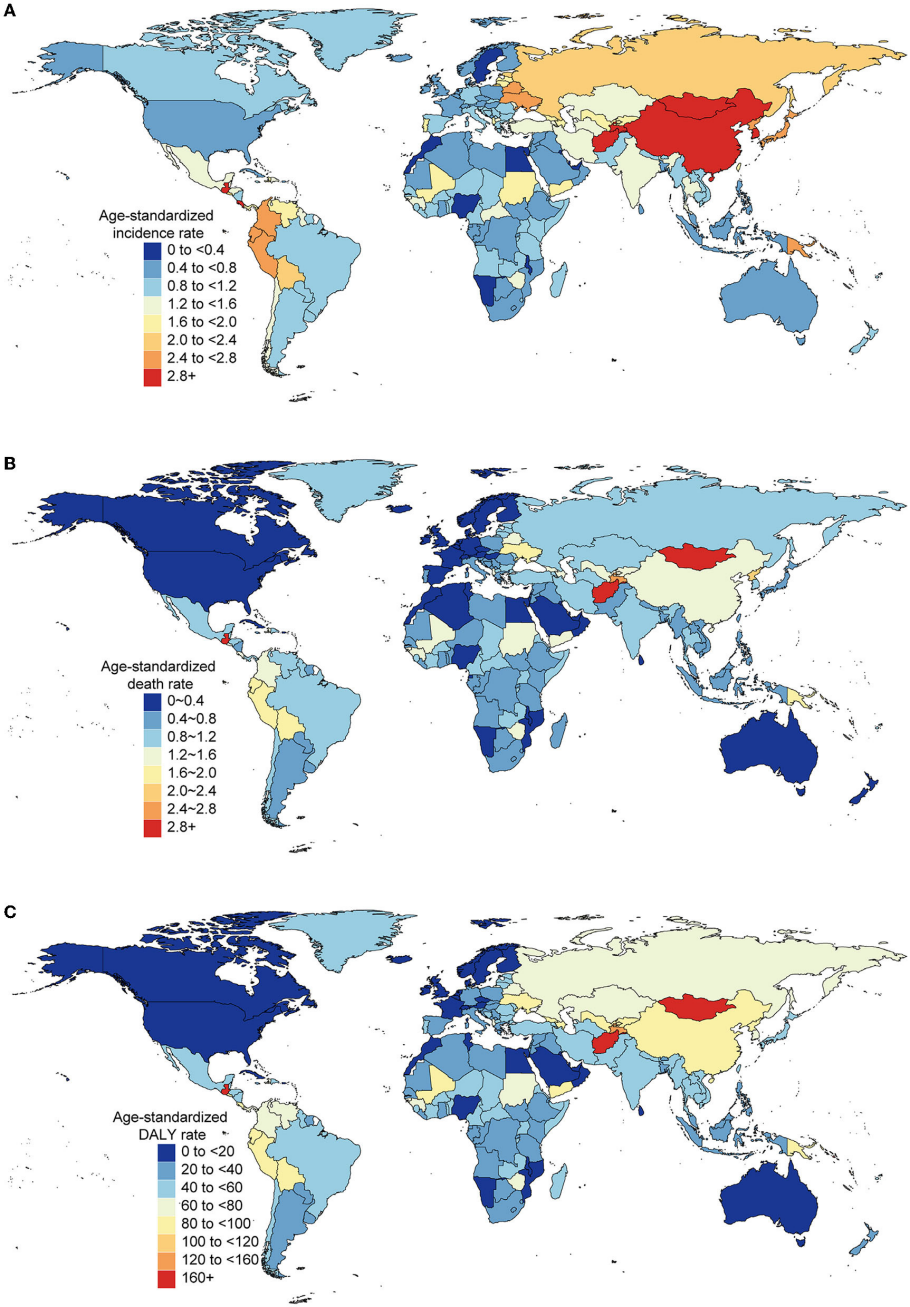
Burden of AYA cancers across 204 countries and territories in both sexes, 2019. Age-standardized rates of incidence **(A)**, death **(B)**, and DALY **(C)** of AYA stomach cancer.

Different countries experienced varying degrees of AYA stomach cancer burden in 2019 (Figures 3A–C) and the associated percentage changes from 1990 to 2019 (Supplementary Figure S6). The highest age-standardized incidence rate occurred in East Asia (3.59 per 100,000 population) and high-income Asia Pacific region (2.88 per 100,000 population; Table 1), particularly in South Korea (3.90 per 100,000 population). China accounted for nearly half of the global incident cases [20,855 (17,648–24,441)] and contributed to 0.47 million (0.41–0.55) DALYs in 2019. Oceania (2.60) and Andean Latin America (2.51) had the subsequent highest age-standardized incidence rates. Three countries outside these high-incidence regions, Afghanistan (3.94), Mongolia (3.48), and Tajikistan (3.03), also had the highest age-standardized incidence rates. The lowest incidence rates were reported in Western sub-Saharan Africa, Australasia, and high-income North America (Table 1).

**Figure 4:**
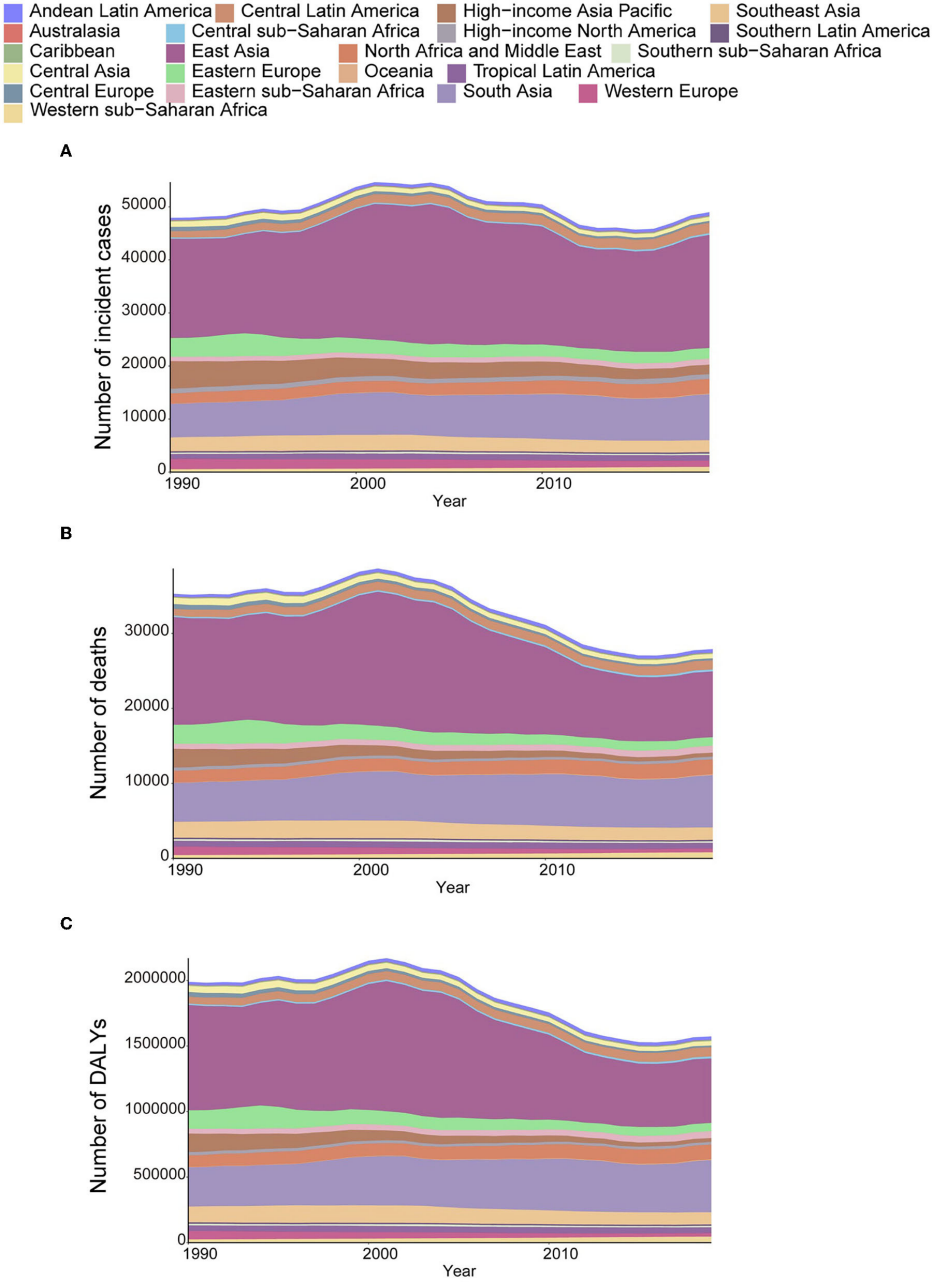
The absolute number of incident cases **(A)**, deaths **(B)**, and DALYs **(C)** due to AYA stomach cancer, 1990–2019, for 21 GBD regions.

In 2019, the number of incident cases of AYA stomach cancer increased from 47,932 (95% UI: 44,593–51,006) to 49,008 (45,008–53,078), an increase of 1,075 cases compared with that reported in 1990. The number of deaths declined from 35,679 (32,579–37,679) to 27,895 (25,711–30,240)-a decrease of 7,784 deaths. The number of DALYs decreased from 1,989,928 (1,839,469–2,123,565) to 1,573,896 (1,448,735–1,703,548)-a decrease of 416,032 DALYs. The highest increase in the number of incidence and decrease in the number of deaths both occurred in East Asia. From the 1990 to 2019, the number of incident cases increased from 18,729 (16,352–21,292) to almost 21,313 (18,107–24,881), but the number of deaths in East Asia decreased in the same period [14,355 (12,466–16,300) in 1990 to 8,749 (7,519–10,098) in 2019]. A major increase in the absolute number of deaths [5,190 (4,459–5,789) in 1990 to 6,981 (6,075–8,025) in 2019] occurred in South Asia with the second highest growth in the number of cases. The bulk of these increases was observed in India. Other regions that contributed significantly to the increased numbers of cases and deaths included central Latin America, North Africa and the Middle East, and Western sub-Saharan Africa (Figures 4A–C).

**Figure 5:**
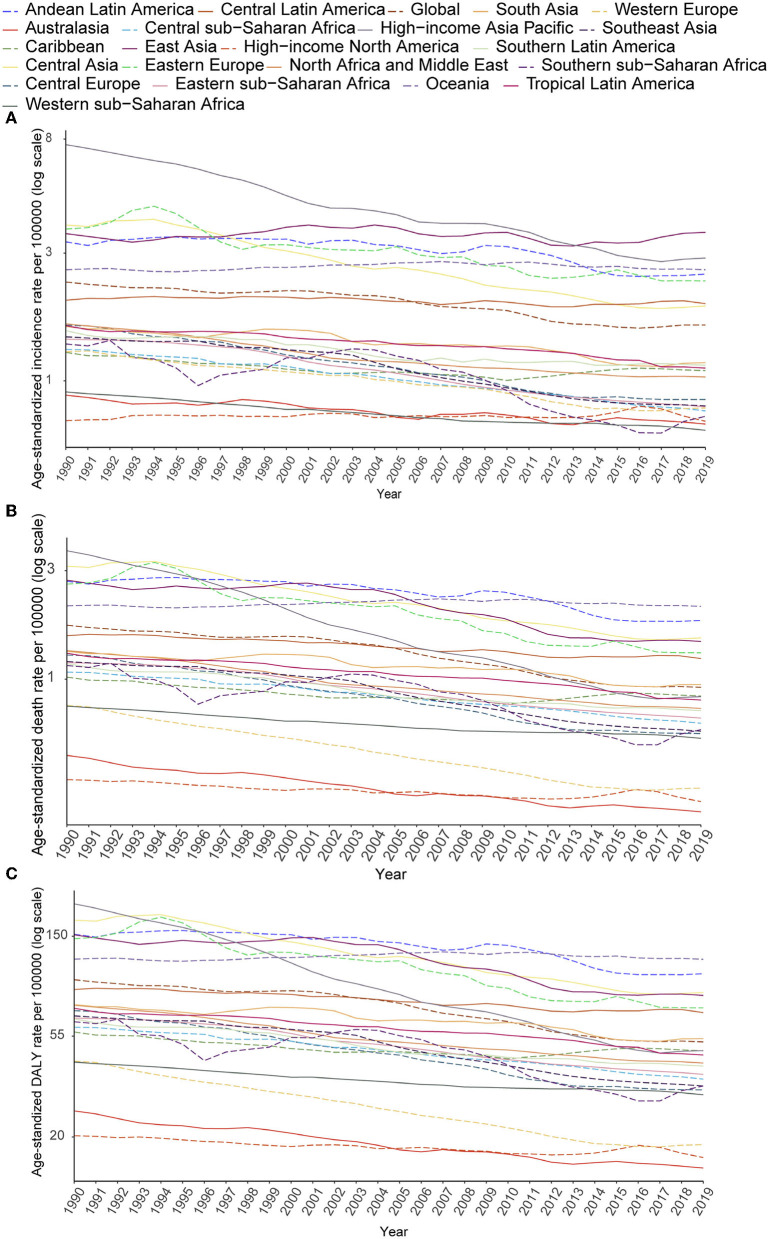
Secular trends of age-standardized incidence **(A)**, death **(B)**, and DALY **(C)** rates of AYA stomach cancer, 1990–2019, globally, and for 21 GBD regions.

Despite the increase in absolute numbers, the global age-standardized incidence and death rates of stomach cancer declined compared with those reported in 1990 (Figures 5A, B). Oceania had the highest age-standardized death rate (2.09), followed by Andean Latin America (1.81) and Central Asia (1.52). The East Asia region, which ranked first in the age-standardized incidence rate, had the fifth highest age-standardized death rate and the fourth highest DALY rate among all GBD regions in 2019. From 1990 to 2019, the incidence, death and DALY rates of AYA stomach cancer decreased in most regions worldwide. However, incidence rates increased in East Asia and high-income North America. The following countries with the highest age-standardized incidence rates also had the highest age-standardized death rates: Afghanistan (3.31), Mongolia (2.93), and Tajikistan (2.50). The lowest age-standardized death rates were reported in high-income North America and Australasia. During this period, the age-standardized incidence rate decreased by 30.90% worldwide. Meanwhile, the age-standardized death rate decreased by 46.77%, and age-standardized DALY declined by 46.43% (Table 1; Figures 5A–C). However, the downward tendency of age-standardized incidence rates did stabilize globally and in some regions during the last 5 years of the study period. At the global level and in many regions, the age-standardized incidence, mortality and DALY rates substantially reduced in women compared with that in men from 1990 to 2019 (Supplementary Figures S7A–C). The high-income Asia Pacific region had the sharpest drop in age-standardized rates between 1990 and 2019 compared with other regions (decrease in age-standardized incidence by 62.27%, age-standardized deaths by 77.25%, and age-standardized DALYs by 77.11%). In East Asia, the age-standardized incidence rate was not sharply reduced (14.7%) and we noticed a 26.55% increase in the age-standardized incidence rate in males (Supplementary Figure S7A). However, a 44.5% decline in age-standardized deaths leads the age-standardized DALY rate to reduce by nearly half (49.7%, Table 1).

### 3.3. Association of AYA stomach cancer with risks and SDI

**Figure 6:**
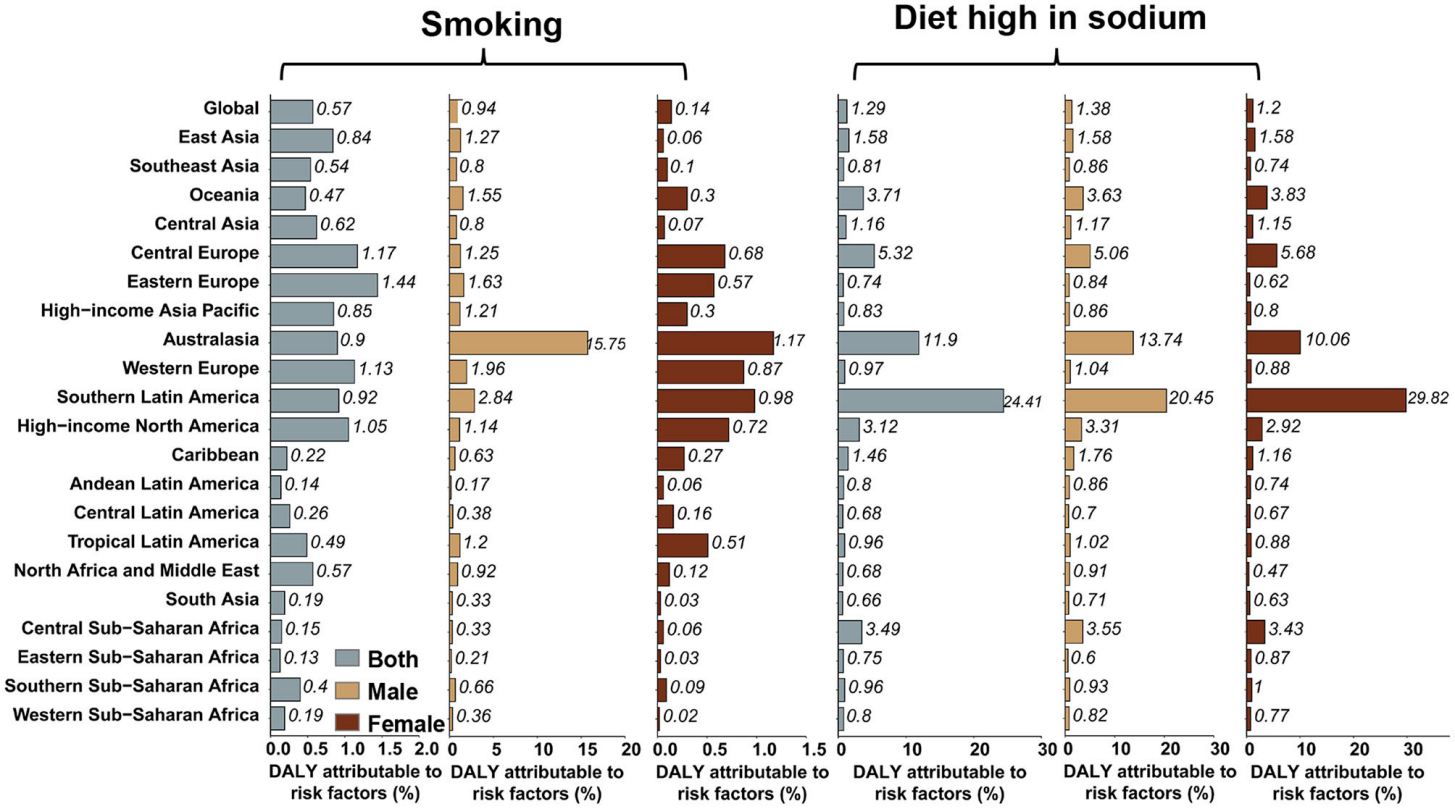
Percentage of stomach cancer age-standardized DALYs attributable to high-sodium diet and smoking in 2019, globally, and for 21 GBD regions.

The percentage of age-standardized DALYs related to the consumption of a high-sodium diet, and the smoking rate in each region are displayed in Figure 6. Globally, 1.29% of age-standardized DALYs were caused by consumption of a high-sodium diet, which was moderately higher in men than in women (1.38% for men; and 1.20% for women). In Southern Latin America, this number was almost twice higher than that of other regions, with 24.41% of age-standardized DALYs being caused by consumption of a high-sodium diet. Globally, 0.57% of age-standardized DALYs were attributed to smoking in men. The highest percentage of age-standardized DALYs attributable to smoking among men was observed in Australasia (15.75%). In women, smoking does not account for a significant proportion of the global age-standardized DALYs. Again, Australasia (more than 1%) had the highest percentage of AYA stomach cancer age-standardized DALYs attributable to smoking among women.

The age-standardized incidence rate of stomach cancer decreased as the SDI increased, and regional diversities of the expected incidence rates progressively magnified as the SDI increased. In contrast to the overall incidence rate of stomach cancer, although the age-standardized incidence rates of AYA stomach cancer decreased as the SDI increased in most regions, they grew in East Asia and high-income North America. But the age-standardized mortality and DALY rates of AYA stomach cancer (Supplementary Figure S8) declined as the SDI increased in all regions.

## 4. Discussion

Although stomach cancer is the main cause of mortality and morbidity in many regions worldwide, the age-standardized incidence and death rates have steadily declined in the previous years. However, the overall trend may mask the critical differences within each age group. The AYA stomach cancer age-standardized incidence and death rates declined slightly in some regions and even increased in East Asia and high-income North America.

Low and middle SDI locations experienced a considerable burden of AYA stomach cancer and more than 40% of these cases were reported in China. A steady decline in death and DALY for stomach cancer has been observed in China over the past several decades. But it is noteworthy that the incidence rate of AYA stomach cancer has still increased in East Asia (especially in China) and the same condition has also occurred in high-income North America.

The present study showed that age-standardized incidence rates did not necessarily follow the changes in age-standardized death and DALY rates; that is, the age-standardized death rates considerably reduced in many locations, while the age-standardized incidence rates did not. East Asia, particularly China, experienced increased age-standardized incidence rates throughout the study period, while the deaths and DALY rates significantly declined. The regional pattern of the AYA stomach cancer burden provides valuable information about its trends and correlates, but the pattern in each region varies greatly. For instance, the AYA stomach cancer incidence rate decreased in Canada but increased in the US. AYA stomach cancer contributes to the higher age-standardized DALY rate per 100,000 population in Portugal, Chile, Ecuador, Guatemala, Bolivia, Yemen, Zimbabwe, Solomon Islands and Mali than in other countries in the same regions. Mongolia, Tajikistan, and Afghanistan have much higher rates than their respective regions (Central Asia, North Africa and Middle East) in terms of age-standardized incidence, death, and DALY rates.

A favorable change in diet and a decrease in *H. pylori* infection rates during childhood may contribute to the decreasing stomach cancer rates in older adults. However, the reasons for the discrepant tendencies in younger people remain unclear. Because of the established relationship between *H. pylori* exposure and stomach cancer, changes in the infection patterns may contribute to the rising rates among younger people. Since early-life infection has been proven to increase the cancer risk (32) and some epidemiological data have demonstrated an association between *H. pylori* infection and increasing risk of stomach cancer among young people (33), the long-term decrease in the prevalence of *H. pylori* could have changed, or the age at infection could have been altered. There is also a possibility that cancers in younger adults may be caused by a new carcinogenic process not properly mediated by *H. pylori* in the gastric mucosa, which may have been unmasked by removing this infection (7). A subgroup of stomach cancer is associated with the Epstein-Barr virus (34), making it possible for these or other agents to play a more significant role in altering gastric microbial flora.

Salt consumption, which has been increasing among all age groups in the US, may also contribute to its occurrence (35). Obesity has been linked to the development of cardiac cancer, and however, it does not seem to increase the risk of non-cardia cancer (36). People have been exposed to hazardous industrial occupational materials during the period of rapid industrialization, especially in developing countries. Since these materials are considered carcinogens, an industrialized workplace environment may play a significant role in the development of stomach cancer at a younger age (37).

A lower incidence of stomach cancer was observed in AYAs with low SDI. In limited-resource settings, cancer incidence is likely to be underestimated owing to misdiagnoses, missed diagnoses, insufficient infrastructure, and weak cancer monitoring systems (38). Global variations in genetic predispositions, lifestyle changes, and environmental exposure may also cause this outcome. A combination of improved reporting and increased exposure to the risk factors may be responsible for the rising global cancer incidence over time. However, there is still a lack of clarity regarding the significance of the interactions between genetics, environment, and non-medical factors.

Given the lack of relevant data, it was difficult to determine the relative contribution of administrative difficulties and other factors to the rapid decline in AYA cancers in South Africa, facing many challenges during the 2000s (39). Monitoring the burden of AYA cancers in the future will reveal the results of the Rwandan Cancer Registry's revitalization in the 2010s (40). The data for Volume X of Cancer Incidence in Five Continents database were obtained from eight cancer registered sites representing only 2% of the total population in Africa. The data surveillance systems in low- and middle-income countries must be strengthened to complete the evaluations based on high-quality cancer data from registered population-based cancer cases.

Our study also has some limitations. Data from some regions were inadequate. We were unable to distinguish the cardia from non-cardia forms from all stomach cancer cases. One of the main reasons for the changing rates of stomach cancer is that non-cardia stomach cancer is predominantly associated with *H. pylori* infection (12). Globally, ~27% of patients with stomach cancers have cardiac tumors (41). The prevalence of cardiac cancer in the US is higher among non-Hispanic whites and is not significantly associated with socioeconomic status (42). There are several challenges in comparing populations and investigating the secular tendency of cardia and non-cardia tumors over time. Because the definition of cardia cancer has changed over time, some cardia tumors might have been classified as lower esophageal adenocarcinomas and vice versa (6). The stomach cancer burden directly caused by *H. pylori* infection has not yet been determined. The lack of data on other risk factors limited our analysis. In addition, details regarding the molecular subtypes of stomach tumors were not obtained. Third, although the GBD 2019 has been updated to include the direct extract rates for those aged 15–39 years, the age-standardized stomach cancer rates for AYA need to be calculated manually rather than extracted directly. Thus, this prevented the estimation of uncertainty intervals, which impeded the assessment of the quality and availability of the source information and statistical uncertainty.

## 5. Conclusion

In this study, AYA stomach cancer appeared to have led to a serious global cancer burden. The overall trend may mask the critical differences among different countries within each age group. Hence, fine-grained analyses should be conducted at the local level to determine the local problems and effective public health policies. Increasing investment in AYA cancer prevention will facilitate equitable access to care and improve the population health in low-income and middle-income countries and worldwide, which will further reduce the number of AYA stomach cancer incident cases and deaths and benefit the younger generation.

## Data availability statement

Publicly available datasets were analyzed in this study. This data can be found here: https://ghdx.healthdata.org.

## Author contributions

Design and analysis: ZZ and JW. Writing—review and editing: ZZ, NS, LS, JW, and JD. Visualization and funding acquisition: ZZ. Supervision: JD. All authors contributed to the article and approved the submitted version.
