# Supplementary material for: The global, regional, and national burden of stomach cancer among adolescents and young adults in 204 countries and territories, 1990–2019: A population-based study
Source: Front Public Health. 2023 Feb 24;11:1079248. doi: 10.3389/fpubh.2023.1079248 (PMC9998989; doi:10.3389/fpubh.2023.1079248)
Supplement: Supplementary file 1 [file Data_Sheet_1.ZIP › supplementary file/Supplementary figures.pdf]

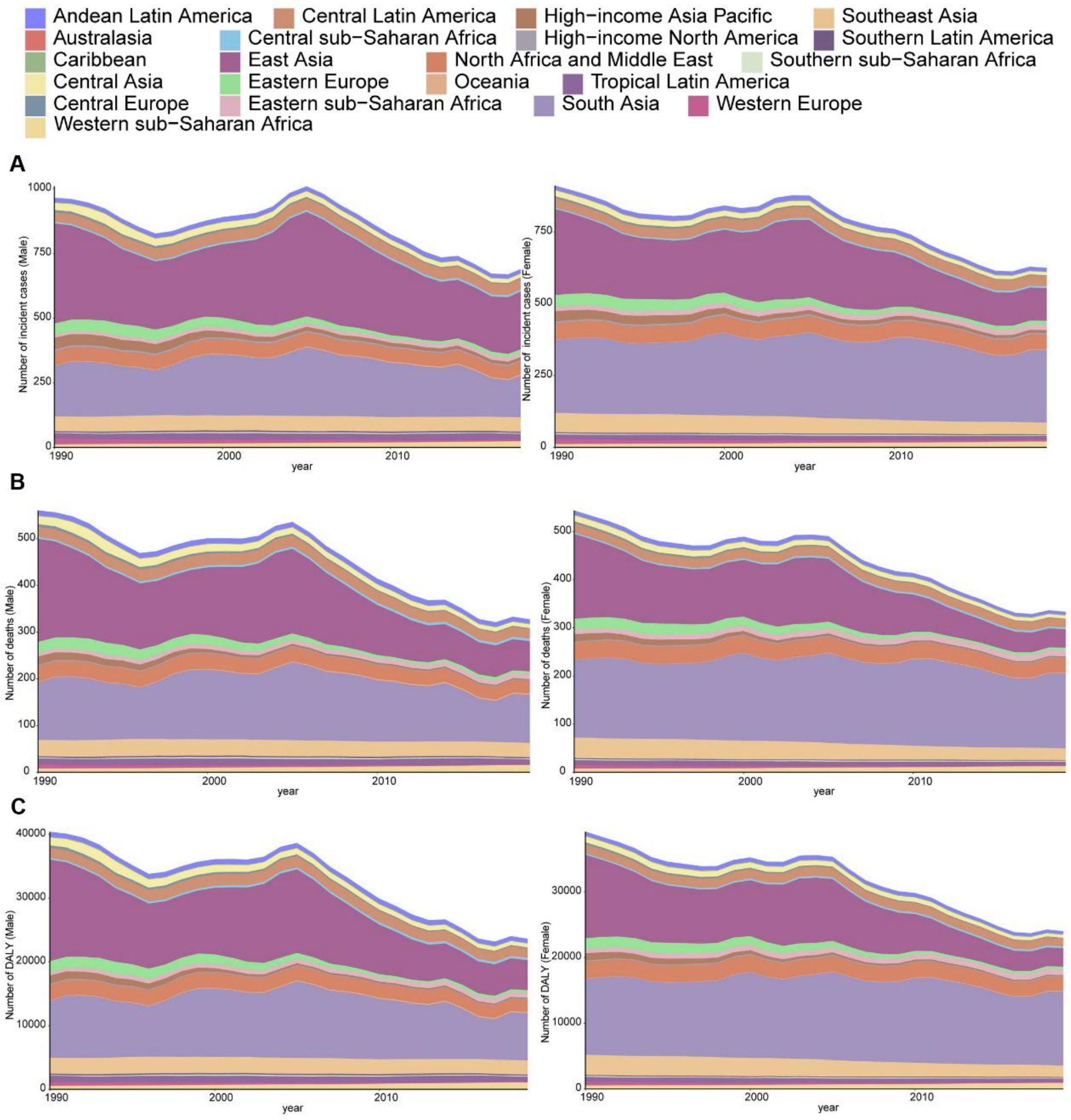

Figure S1: Global trends in the absolute number of incident cases, deaths, and DALYs of AYA stomach cancer by sex among 15- to 19-year-olds, 1990–2019. (A) Trends in the number of incident cases of AYA stomach cancer. (B) Trends in the number of deaths of AYA stomach cancer. (C) Trends in the number of DALYs of AYA stomach cancer.

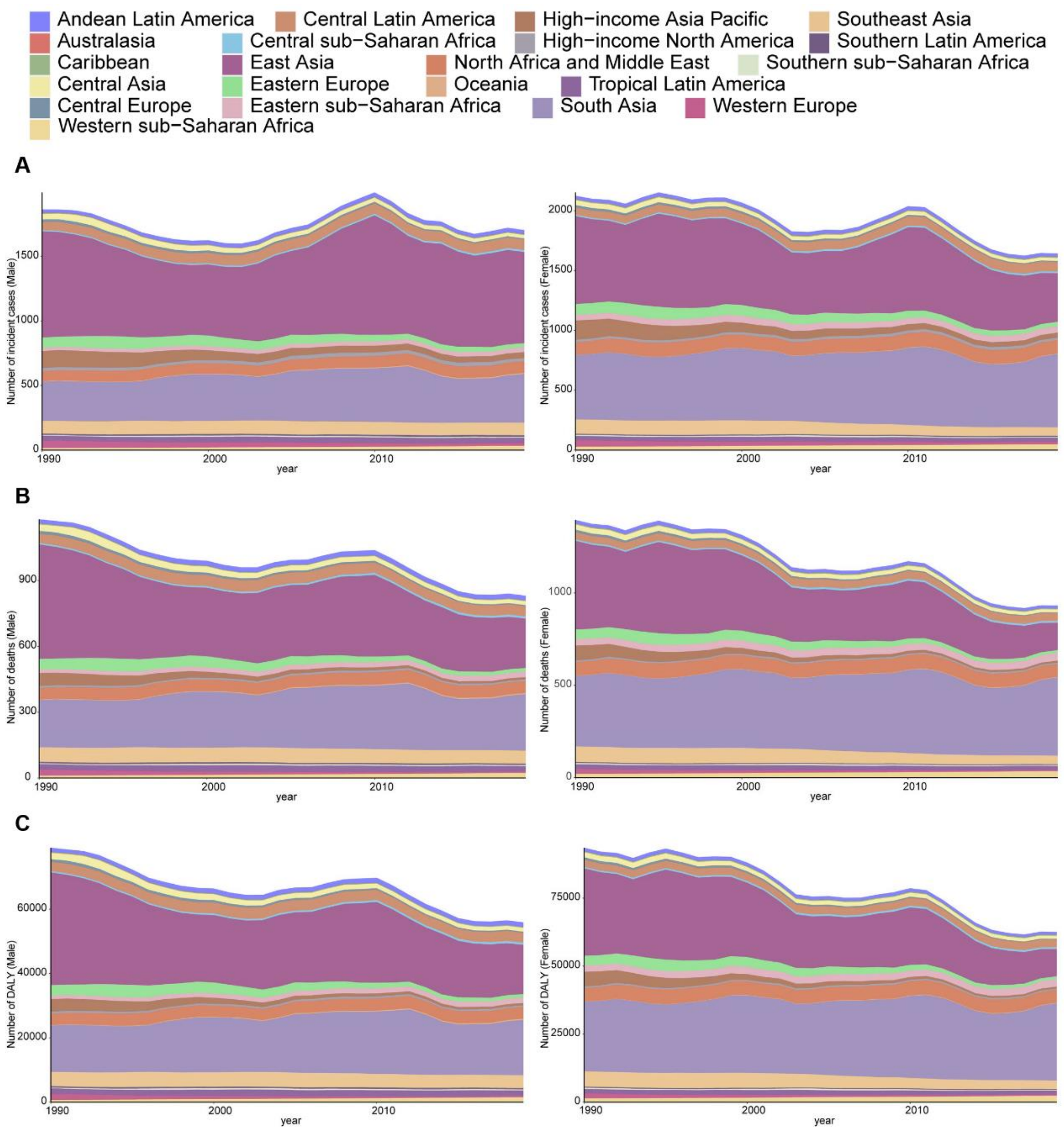

Figure S2: Global trends in the absolute number of incident cases, deaths, and DALYs of AYA stomach cancer by sex among 20- to 24-year-olds, 1990–2019. (A) Trends in the number of incident cases of AYA stomach cancer. (B) Trends in the number of deaths of AYA stomach cancer. (C) Trends in the number of DALYs of AYA stomach cancer.

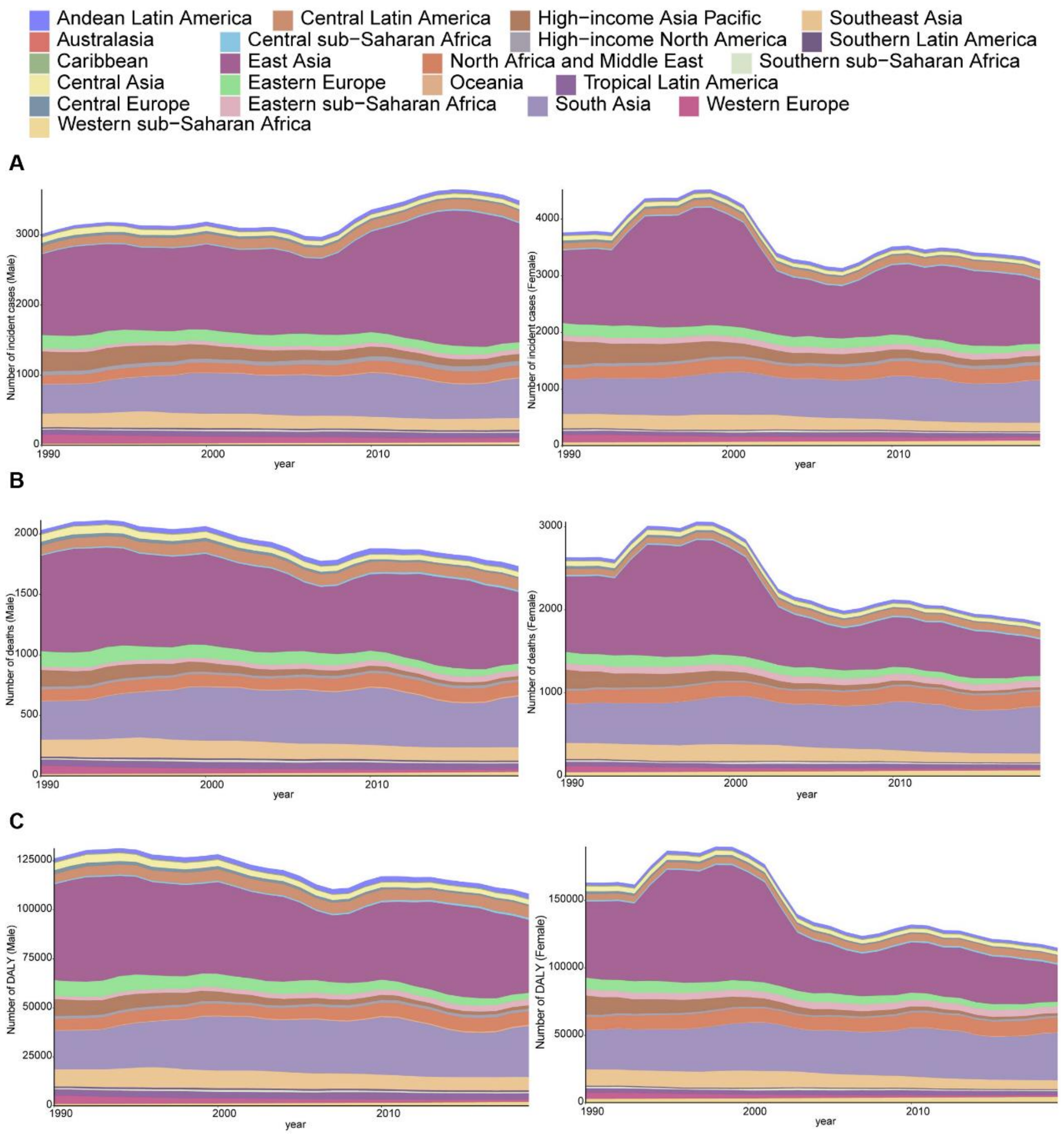

Figure S3: Global trends in the absolute number of incident cases, deaths, and DALYs of AYA stomach cancer by sex among 25- to 29-year-olds, 1990–2019. (A) Trends in the number of incident cases of AYA stomach cancer. (B) Trends in the number of deaths of AYA stomach cancer. (C) Trends in the number of DALYs of AYA stomach cancer.

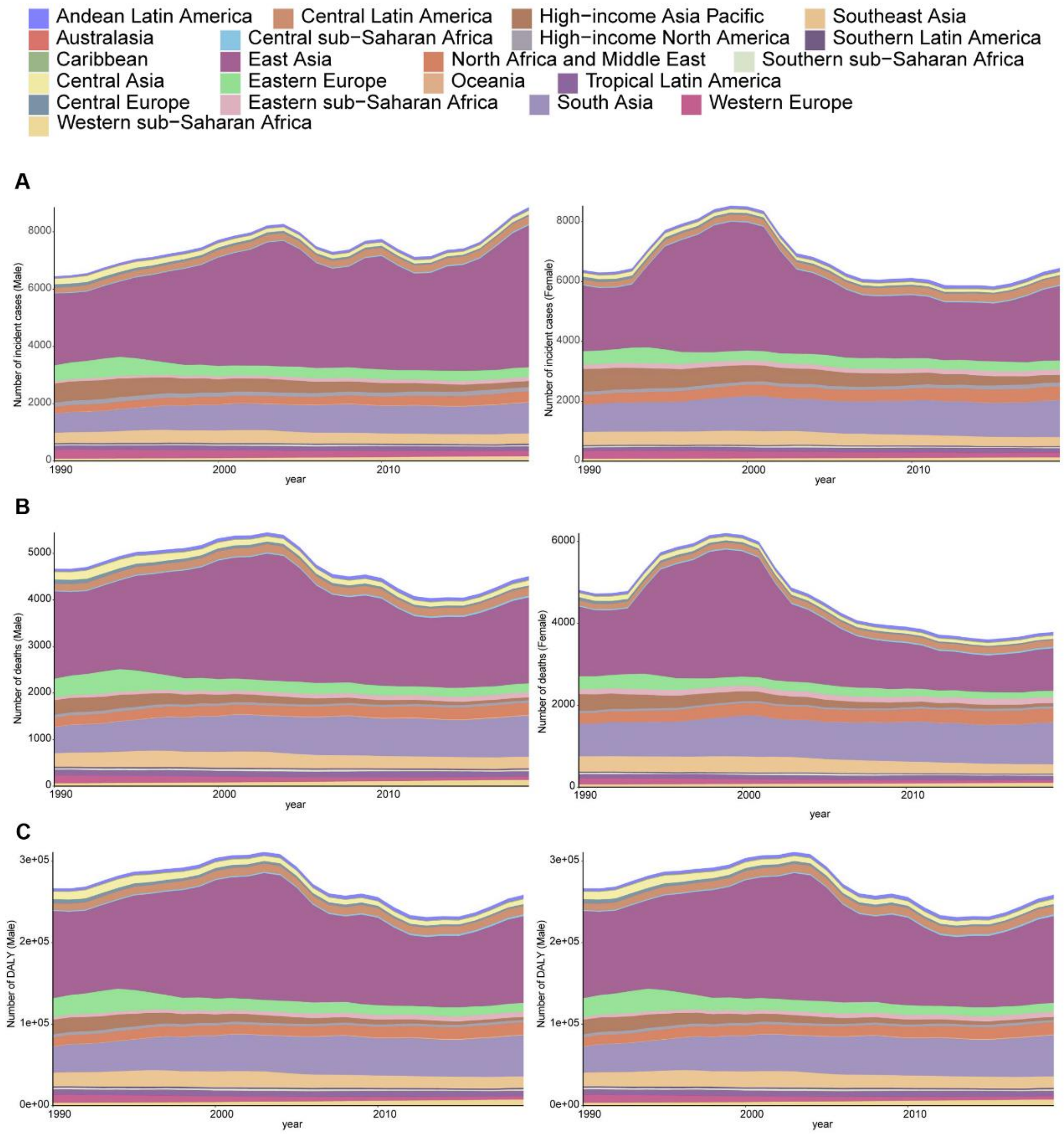

Figure S4: Global trends in the absolute number of incident cases, deaths, and DALYs of AYA stomach cancer by sex among 30- to 34-year-olds, 1990–2019. (A) Trends in the number of incident cases of AYA stomach cancer. (B) Trends in the number of deaths of AYA stomach cancer. (C) Trends in the number of DALYs of AYA stomach cancer.

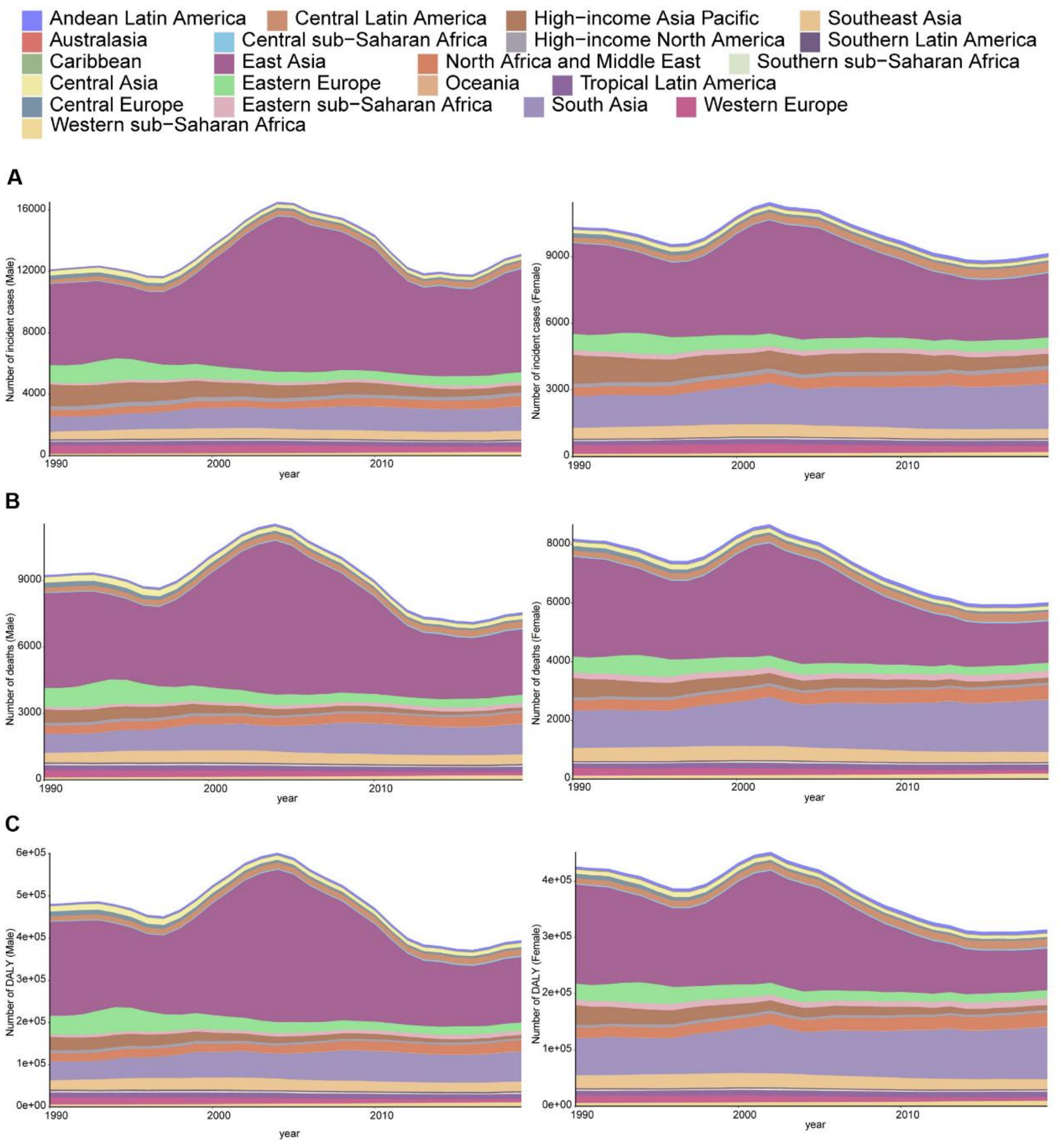

Figure S5: Global trends in the absolute number of incident cases, deaths, and DALYs of AYA stomach cancer by sex among 35- to 39-year-olds, 1990–2019. (A) Trends in the number of incident cases of AYA stomach cancer. (B) Trends in the number of deaths of AYA stomach cancer. (C) Trends in the number of DALYs of AYA stomach cancer.

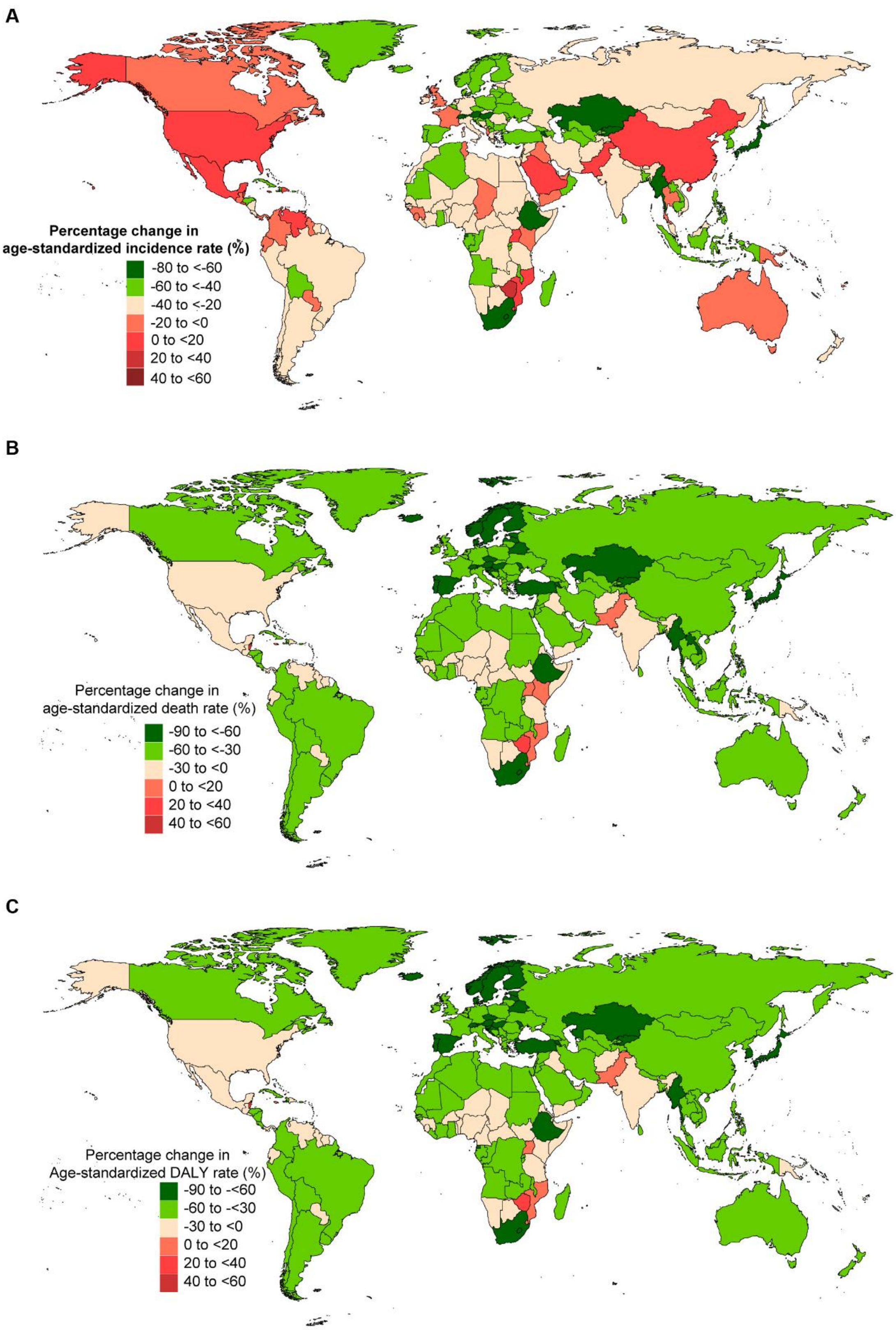

Figure S6: The percentage change of AYA stomach cancer across 204 countries and territories in both sexes, 2019.The percentage change in age-standardized incidence (A), death (B), and DALY (C) rates of AYA stomach cancer in both sexes from 1990 to 2019.

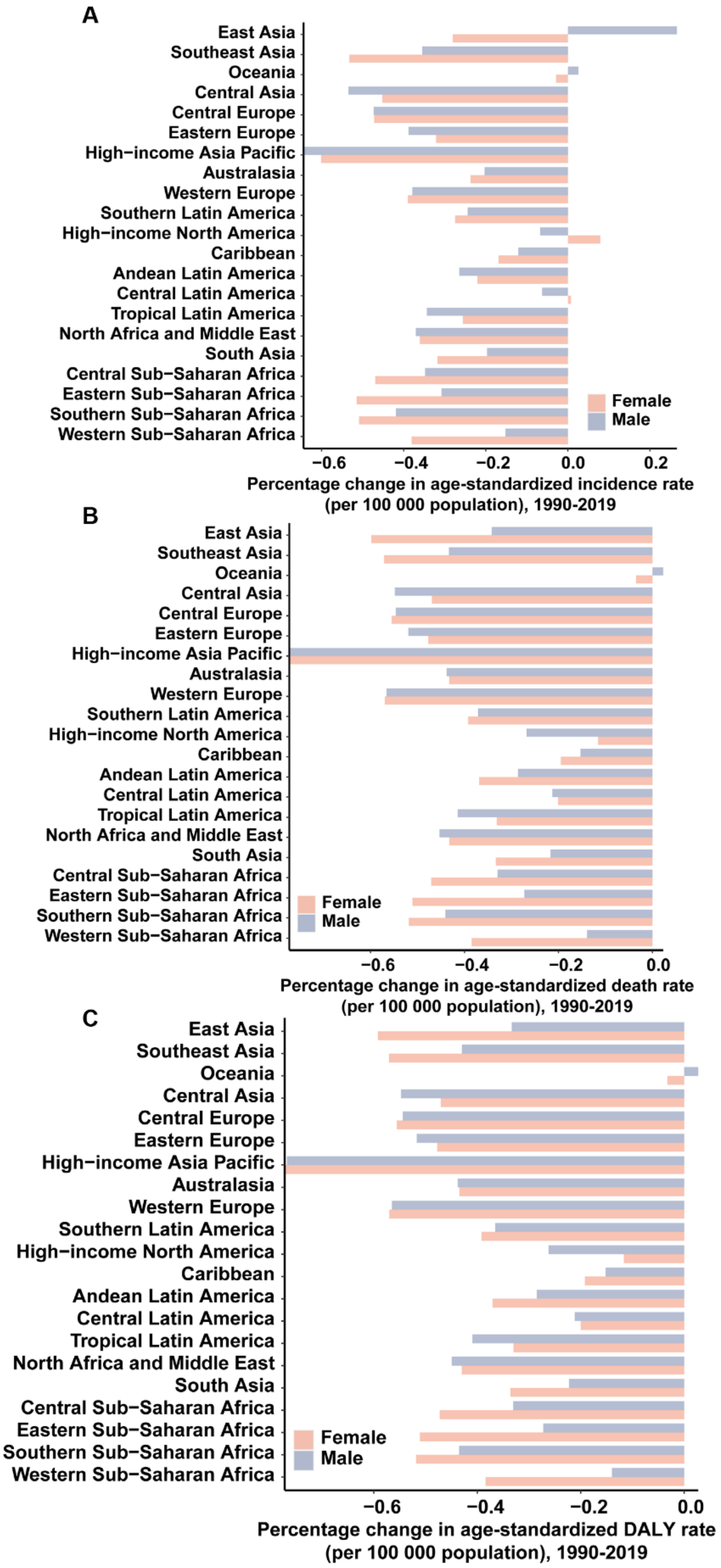

Figure S7: Trends in the burden of AYA stomach cancer across 21 GBD regions by sex. The percentage change in age-standardized incidence (A), death (B), and DALY (C) rates of AYA stomach cancer from 1990 to 2019. GBD=Global Burden of Diseases, Injuries, and Risk Factors Study

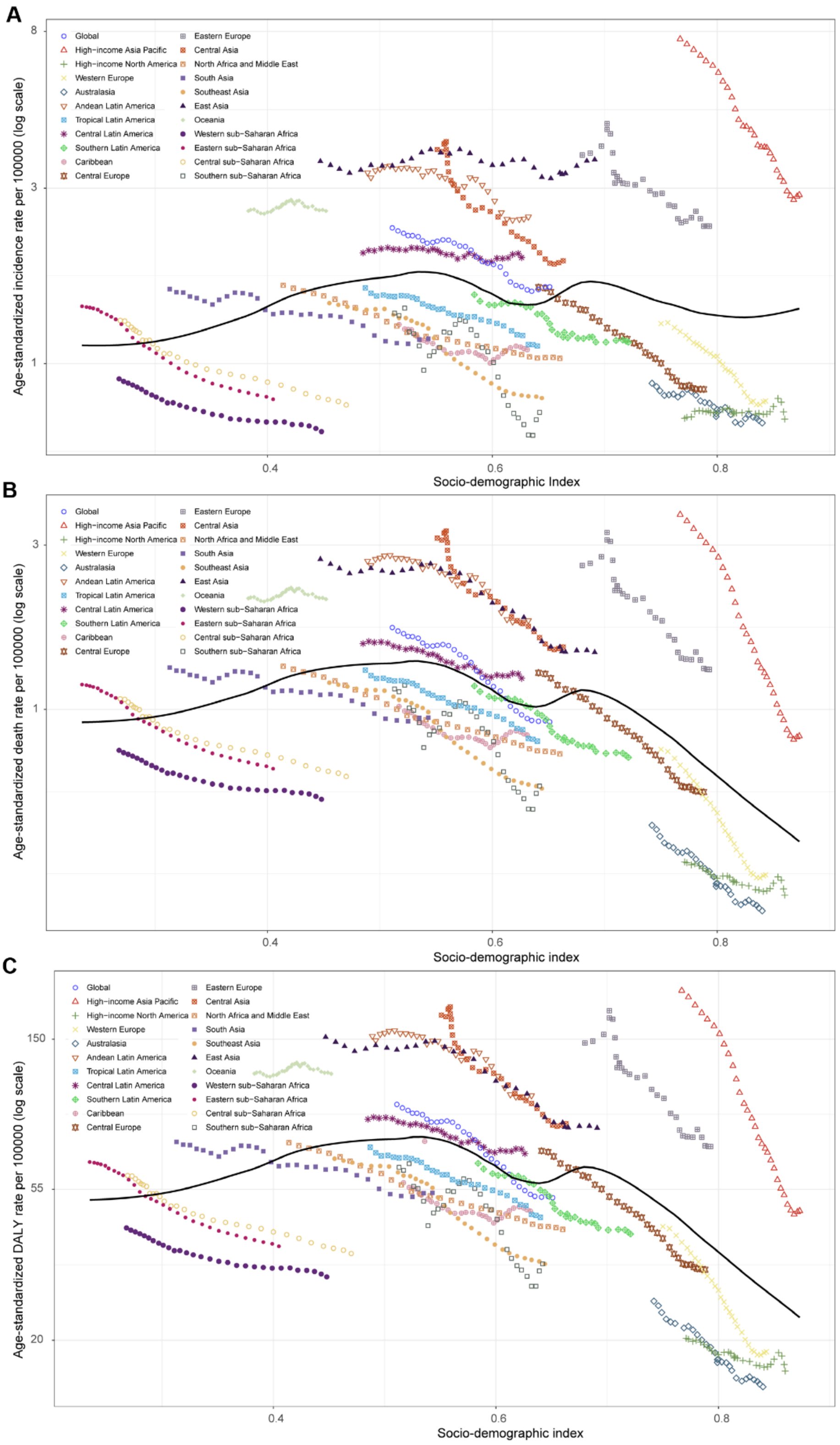

Figure S8: Association between SDI and AYA stomach cancer burden at the national level in 2019. The association between SDI and the age-standardized incidence rate (A), death rate (B), and DALY rate (C) are depicted. For each region, points from left to right depict estimates from each year from 1990 to 2019. Lines are based on fitted regression models.
