# Supplementary material for: The global, regional, and national burden of stomach cancer among adolescents and young adults in 204 countries and territories, 1990–2019: A population-based study
Source: Front Public Health. 2023 Feb 24;11:1079248. doi: 10.3389/fpubh.2023.1079248 (PMC9998989; doi:10.3389/fpubh.2023.1079248)
Supplement: Supplementary file 1 [file Data_Sheet_1.ZIP › supplementary file/Supplementary tables.pdf]

Table S1: Incident cases, deaths, and DALYs of AYA cancers among 15 to 19 years in 2019, and percentage change in age-specific rates from 1990 to 2019, by sex, SDI quintile, and cancer types.

	**Incidence**			**Deaths**			**DALYs**		
	**Number of incident cases**	**Age-specific incidence rate (per 100 000 population)**	**APC**	**Number of deaths**	**Age-specific death rate (per 100 000 population)**	**APC**	**Number of DALYs**	**Age-specific DALYs rate (per 100 000 population)**	**APC**
Global	1315(1176-1456)	0.21(0.19-0.23)	-1.84(-2.2 to -1.48)*	661(593-740)	0.11(0.10-0.12)	-2.32(-2.77 to -1.87)*	47627(42821-53211)	7.69(6.91-8.59)	-2.32(-2.77 to -1.87)*
Male	690(602-787)	0.22(0.19-0.25)	-1.77(-2.29 to -1.23)*	327(288-378)	0.10(0.09-0.12)	-2.42(-3.03 to -1.80)*	23619(20777-27221)	7.43(6.54-8.57)	-2.41(-3.03 to -1.80)*
Female	625(553-707)	0.21(0.18-0.23)	-1.86(-2.28 to -1.43)*	333(290-382)	0.11(0.10-0.13)	-2.26(-2.72 to -1.79)*	24008(20878-27518)	7.96(6.92-9.12)	-2.26(-2.72 to -1.79)*
East Asia	336(286-397)	0.43(0.37-0.51)	-0.72(-1.08 to -0.36)*	103(88-119)	0.13(0.11-0.15)	-2.88(-3.06 to -2.69)*	7453(6405-8642)	9.53(8.19-11.05)	-2.85(-3.03 to -2.66)*
Southeast Asia	97(81-113)	0.17(0.14-0.20)	-1.34(-1.5 to -1.18)*	55(46-65)	0.10(0.08-0.11)	-1.63(-1.77 to -1.49)*	3994(3342-4664)	7.05(5.9-8.23)	-1.63(-1.77 to -1.49)*
Oceania	7(5-11)	0.57(0.39-0.85)	0.34(0.11 to 0.57)*	5(3-7)	0.35(0.24-0.52)	0.31(0.09 to 0.54)*	326(219-475)	25.49(17.14-37.18)	0.31(0.09 to 0.54)*
Central Asia	24(21-28)	0.34(0.30-0.39)	-2.25(-3.1 to -1.4)*	15(13-17)	0.21(0.18-0.24)	-2.37(-3.19 to -1.53)*	1054(914-1216)	14.87(12.9-17.16)	-2.36(-3.20 to -1.52)*
Central Europe	5(4-6)	0.09(0.07-0.10)	-2.18(-2.7 to -1.66)*	3(2-3)	0.04(0.04-0.05)	-2.69(-3.22 to -2.16)*	185(153-219)	3.22(2.66-3.81)	-2.69(-3.22 to -2.16)*
Eastern Europe	27(23-32)	0.26(0.22-0.31)	-2.59(-4.28 to -0.87)*	12(10-15)	0.11(0.09-0.14)	-3.27(-5.01 to -1.5)*	858(709-1060)	8.28(6.84-10.22)	-3.26(-5.00 to -1.49)*
High-income Asia Pacific	21(17-25)	0.23(0.19-0.28)	-2.79(-3.47 to -2.11)*	4(4-5)	0.05(0.04-0.06)	-5.05(-5.66 to -4.43)*	313(275-364)	3.5(3.07-4.07)	-5.03(-5.63 to -4.43)*
Australasia	1(1-1)	0.06(0.04-0.08)	-1.84(-2.96 to -0.71)*	0(0-0)	0.02(0.01-0.02)	-2.97(-3.99 to -1.94)*	21(16-27)	1.19(0.93-1.56)	-2.94(-3.97 to -1.91)*
Western Europe	15(13-18)	0.06(0.05-0.08)	-2.9(-3.38 to -2.43)*	5(4-5)	0.02(0.02-0.02)	-4.00(-4.43 to -3.58)*	336(302-376)	1.43(1.29-1.61)	-3.98(-4.41 to -3.56)*
Southern Latin America	8(6-12)	0.17(0.11-0.24)	-0.44(-0.81 to -0.06)*	4(3-6)	0.08(0.06-0.11)	-1.02(-1.49 to -0.55)*	294(216-402)	5.81(4.26-7.93)	-1.02(-1.49 to -0.54)*
High-income North America	12(10-14)	0.05(0.04-0.06)	-1.26(-1.82 to -0.70)*	4(3-4)	0.02(0.01-0.02)	-2.04(-2.50 to -1.58)*	264(243-288)	1.12(1.03-1.22)	-2.03(-2.49 to -1.57)*
Caribbean	7(5-9)	0.18(0.13-0.24)	-0.45(-0.66 to -0.24)*	4(3-6)	0.11(0.08-0.15)	-0.53(-0.75 to -0.30)*	301(212-403)	7.82(5.52-10.47)	-0.53(-0.75 to -0.3)*
Andean Latin America	25(19-34)	0.46(0.34-0.61)	-1.35(-1.81 to -0.89)*	14(10-19)	0.25(0.19-0.34)	-1.70(-2.13 to -1.26)*	1019(749-1373)	18.23(13.41-24.56)	-1.7(-2.13 to -1.26)*
Central Latin America	76(63-89)	0.34(0.29-0.40)	-0.24(-0.34 to -0.14)*	37(31-44)	0.17(0.14-0.2)	-0.89(-0.99 to -0.79)*	2699(2253-3206)	12.23(10.21-14.53)	-0.88(-0.98 to -0.78)*
Tropical Latin America	31(28-35)	0.18(0.16-0.20)	-0.98(-1.77 to -0.19)*	17(15-19)	0.10(0.09-0.11)	-1.36(-2.15 to -0.56)*	1224(1102-1360)	7.16(6.44-7.95)	-1.35(-2.14 to -0.56)*
North Africa and Middle East	100(83-122)	0.19(0.16-0.23)	-1.63(-1.83 to -1.42)*	55(45-68)	0.10(0.09-0.13)	-1.95(-2.15 to -1.75)*	3997(3249-4920)	7.55(6.14-9.29)	-1.95(-2.15 to -1.75)*
South Asia	420(343-507)	0.24(0.19-0.29)	-2.33(-3.00 to -1.66)*	259(212-310)	0.15(0.12-0.17)	-2.09(-3.68 to -0.49)*	18645(15288-22259)	10.49(8.6-12.52)	-2.10(-3.68 to -0.49)*
Central sub-Saharan Africa	16(11-21)	0.11(0.08-0.15)	-2.08(-2.18 to -1.98)*	10(7-13)	0.07(0.05-0.1)	-2.10(-2.20 to -2.00)*	719(494-968)	5.09(3.5-6.86)	-2.10(-2.2 to -2.01)*
Eastern sub-Saharan Africa	36(29-44)	0.08(0.06-0.09)	-1.83(-1.93 to -1.73)*	23(19-28)	0.05(0.04-0.06)	-1.81(-1.9 to -1.71)*	1678(1362-2050)	3.57(2.9-4.36)	-1.81(-1.9 to -1.71)*
Southern sub-Saharan Africa	5(4-7)	0.08(0.06-0.10)	-0.94(-1.35 to -0.52)*	3(3-4)	0.05(0.04-0.06)	-1.21(-1.81 to -0.61)*	238(184-307)	3.4(2.63-4.39)	-1.21(-1.81 to -0.61)*
Western sub-Saharan Africa	44(34-56)	0.09(0.07-0.11)	-1.07(-1.21 to -0.92)*	28(21-35)	0.06(0.04-0.07)	-1.09(-1.23 to -0.95)*	2009(1534-2521)	4.02(3.07-5.05)	-1.09(-1.23 to -0.95)*

Table S2: Incident cases, deaths, and DALYs of AYA cancers among 20 to 24 years in 2019, and percentage change in age-specific rates from 1990 to 2019, by sex, SDI quintile, and cancer types.

	**Incidence**			**Deaths**			**DALYs**		
	**Number of incident cases**	**Age-specific incidence rate (per 100 000 population)**	**APC**	**Number of deaths**	**Age-specific death rate (per 100 000 population)**	**APC**	**Number of DALYs**	**Age-specific DALYs rate (per 100 000 population)**	**APC**
Global	3345(3043-3652)	0.56(0.51-0.61)	-1.26(-1.54 to -0.98)*	1763(1599-1944)	0.29(0.27-0.32)	-1.95(-2.19 to -1.72)*	118474(107533-130706)	19.74(17.92-21.78)	-1.95(-2.18 to -1.71)*
Male	1703(1503-1915)	0.56(0.49-0.63)	-1.00(-1.27 to -0.73)*	831(746-930)	0.27(0.25-0.31)	-1.89(-2.09 to -1.68)*	55907(50207-62551)	18.37(16.50-20.56)	-1.88(-2.09 to -1.67)*
Female	1642(1447-1869)	0.56(0.49-0.63)	-1.50(-1.90 to -1.11)*	932(816-1060)	0.32(0.28-0.36)	-2.00(-2.39 to -1.60)*	62567(54815-71168)	21.15(18.53-24.06)	-1.99(-2.38 to -1.60)*
East Asia	1109(932-1312)	1.30(1.09-1.54)	0.65(-0.15 to 1.45)*	372(317-436)	0.44(0.37-0.51)	-1.60(-2.30 to -0.88)*	25228(21508-29490)	29.53(25.18-34.52)	-1.56(-2.26 to -0.86)*
Southeast Asia	168(143-195)	0.30(0.26-0.35)	-1.66(-1.85 to -1.47)*	106(90-121)	0.19(0.16-0.22)	-1.99(-2.19 to -1.79)*	7080(6070-8097)	12.72(10.91-14.55)	-1.98(-2.18 to -1.79)*
Oceania	14(9-19)	1.13(0.77-1.58)	0.16(-0.12 to 0.44)*	9(6-13)	0.77(0.53-1.09)	0.15(-0.11 to 0.40)	634(435-887)	51.9(35.64-72.68)	0.15(-0.11 to 0.4)
Central Asia	51(44-60)	0.68(0.59-0.79)	-2.42(-2.95 to -1.89)*	34(30-40)	0.46(0.39-0.53)	-2.51(-3.03 to -1.99)*	2308(1985-2685)	30.67(26.38-35.67)	-2.51(-3.03 to -1.99)*
Central Europe	15(13-18)	0.25(0.21-0.29)	-2.26(-2.70 to -1.81)*	9(7-10)	0.14(0.12-0.16)	-2.74(-3.23 to -2.25)*	580(494-676)	9.43(8.03-11)	-2.73(-3.22 to -2.24)*
Eastern Europe	69(60-79)	0.67(0.58-0.76)	-1.63(-2.33 to -0.93)*	33(28-39)	0.32(0.27-0.38)	-2.42(-3.64 to -1.2)*	2219(1886-2621)	21.54(18.31-25.45)	-2.42(-3.63 to -1.19)*
High-income Asia Pacific	88(73-103)	0.89(0.74-1.05)	-2.98(-3.38 to -2.59)*	20(18-23)	0.21(0.19-0.23)	-5.38(-5.77 to -4.98)*	1389(1253-1547)	14.06(12.69-15.66)	-5.33(-5.72 to -4.93)*
Australasia	4(3-5)	0.20(0.15-0.27)	-1.54(-2.22 to -0.85)*	1(1-1)	0.06(0.05-0.08)	-2.74(-3.36 to -2.11)*	79(61-99)	4.18(3.27-5.27)	-2.72(-3.34 to -2.09)*
Western Europe	45(38-53)	0.18(0.16-0.22)	-2.46(-2.85 to -2.06)*	16(14-17)	0.06(0.06-0.07)	-3.61(-4.06 to -3.17)*	1061(966-1168)	4.32(3.94-4.76)	-3.59(-4.04 to -3.15)*
Southern Latin America	20(14-27)	0.38(0.27-0.53)	-0.69(-1.13 to -0.26)*	11(8-13)	0.2(0.16-0.26)	-1.19(-1.61 to -0.78)*	708(556-899)	13.57(10.66-17.24)	-1.18(-1.60 to -0.77)*
High-income North America	46(40-54)	0.19(0.17-0.23)	-0.27(-1.03 to 0.50)*	15(14-17)	0.07(0.06-0.07)	-1.06(-1.67 to -0.46)*	1048(974-1132)	4.4(4.09-4.76)	-1.05(-1.66 to -0.44)*
Caribbean	14(10-18)	0.37(0.27-0.48)	-0.37(-0.63 to -0.11)*	9(7-12)	0.24(0.17-0.32)	-0.44(-0.68 to -0.19)*	622(444-810)	16.38(11.69-21.34)	-0.44(-0.68 to -0.19)*
Andean Latin America	59(44-76)	1.06(0.79-1.37)	-1.08(-1.74 to -0.41)*	36(27-47)	0.65(0.49-0.85)	-1.47(-2.11 to -0.82)*	2429(1809-3141)	43.84(32.66-56.7)	-1.47(-2.11 to -0.82)*
Central Latin America	161(133-195)	0.74(0.61-0.89)	-0.10(-0.36 to 0.17)*	87(72-105)	0.4(0.33-0.48)	-0.78(-1.04 to -0.52)*	5859(4856-7042)	27(22.38-32.44)	-0.78(-1.04 to -0.52)*
Tropical Latin America	73(67-80)	0.40(0.37-0.44)	-0.68(-1.34 to -0.01)*	44(40-48)	0.24(0.22-0.27)	-1.03(-1.60 to -0.45)*	2971(2716-3247)	16.41(15-17.93)	-1.04(-1.71 to -0.36)*
North Africa and Middle East	202(165-250)	0.39(0.32-0.48)	-1.31(-1.46 to -1.17)*	124(99-156)	0.24(0.19-0.3)	-1.69(-1.85 to -1.53)*	8332(6639-10479)	16.11(12.83-20.26)	-1.69(-1.84 to -1.53)*
South Asia	987(830-1168)	0.59(0.49-0.69)	-1.38(-1.97 to -0.77)*	680(569-808)	0.4(0.34-0.48)	-1.46(-2.12 to -0.79)*	45565(38133-54017)	27.07(22.65-32.09)	-1.46(-2.13 to -0.79)*
Central Sub-Saharan Africa	31(22-40)	0.26(0.19-0.34)	-1.85(-1.95 to -1.76)*	22(16-28)	0.18(0.13-0.24)	-1.83(-1.92 to -1.73)*	1441(1039-1886)	12.09(8.72-15.82)	-1.83(-1.92 to -1.73)*
Eastern Sub-Saharan Africa	93(75-117)	0.24(0.19-0.29)	-2.05(-2.23 to -1.87)*	66(53-83)	0.17(0.14-0.21)	-1.98(-2.11 to -1.85)*	4414(3580-5534)	11.28(9.15-14.15)	-1.98(-2.11 to -1.85)*
Southern Sub-Saharan Africa	14(11-18)	0.21(0.16-0.26)	-1.41(-2.04 to -0.77)*	10(8-12)	0.14(0.11-0.18)	-1.46(-2.09 to -0.83)*	641(507-803)	9.41(7.44-11.8)	-1.46(-2.08 to -0.82)*
Western Sub-Saharan Africa	82(64-103)	0.20(0.15-0.25)	-1.11(-1.25 to -0.98)*	58(44-72)	0.14(0.11-0.17)	-1.11(-1.24 to -0.98)*	3869(2979-4838)	9.32(7.18-11.66)	-1.11(-1.24 to -0.98)*

TableS3: Incident cases, deaths, and DALYs of AYA cancers among 25 to 29 years in 2019, and percentage change in age-specific rates from 1990 to 2019, by sex, and cancer types.

	Incidence			Deaths			DALYs		
	**Number of incident cases**	**Age-specific incidence rate (per 100 000 population)**	**APC**	**Number of deaths**	**Age-specific death rate (per 100 000 population)**	**APC**	**Number of DALYs**	**Age-specific DALYs rate (per 100 000 population)**	**APC**
Global	6739(6135-7336)	1.11(1.01-1.21)	-1.02(-1.52 to -0.52)*	3581(3271-3900)	0.59(0.54-0.64)	-1.89(-2.49 to -1.29)*	223068(204190-242918)	36.84(33.72-40.12)	-1.88(-2.48 to -1.28)*
Male	3489(3151-3926)	1.14(1.03-1.29)	-0.56(-0.88 to -0.23)*	1735(1584-1924)	0.57(0.52-0.63)	-1.71(-2.12 to -1.19)*	108203(99118-119906)	35.50(32.52-39.34)	-1.69(-2.10 to -1.27)
Female	3249(2838-3696)	1.08(0.94-1.23)	-1.51(-2.39 to -0.62)	1847(1630-2086)	0.61(0.54-0.69)	-2.27(-3.24 to -1.29)*	114865(101513-129666)	38.20(33.76-43.12)	-2.26(-3.23 to -1.27)*
East Asia	2814(2375-3313)	2.46(2.07-2.89)	0.43(-0.59 to 1.45)	1031(877-1191)	0.9(0.77-1.04)	-1.55(-2.47 to -0.62)*	64683(55229-74782)	56.46(48.21-65.28)	-1.51(-2.43 to -0.58)*
Southeast Asia	322(271-375)	0.58(0.49-0.68)	-2.2(-2.38 to -2.02)*	217(183-252)	0.39(0.33-0.46)	-2.55(-2.73 to -2.37)*	13456(11364-15627)	24.43(20.64-28.38)	-2.55(-2.72 to -2.37)*
Oceania	26(18-36)	2.36(1.59-3.26)	0.07(-0.04 to 0.17)	19(13-27)	1.75(1.18-2.41)	0.08(-0.02 to 0.18)	1204(815-1654)	108.57(73.45-149.15)	0.08(-0.02 to 0.18)
Central Asia	108(90-128)	1.28(1.08-1.52)	-2.19(-2.64 to -1.73)*	79(66-93)	0.94(0.78-1.11)	-2.29(-2.77 to -1.82)*	4868(4080-5767)	57.98(48.59-68.69)	-2.29(-2.77 to -1.82)*
Central Europe	41(34-48)	0.57(0.48-0.68)	-2.25(-2.78 to -1.71)*	25(21-29)	0.35(0.3-0.41)	-2.81(-3.33 to -2.28)*	1540(1305-1812)	21.64(18.34-25.46)	-2.80(-3.32 to -2.27)*
Eastern Europe	204(175-236)	1.53(1.31-1.77)	-1.25(-2.1 to -0.39)*	104(90-121)	0.78(0.67-0.91)	-2.05(-2.81 to -1.27)*	6465(5581-7527)	48.44(41.82-56.4)	-2.04(-2.81 to -1.26)*
High-income Asia Pacific	204(175-240)	1.97(1.68-2.32)	-3.45(-4.22 to -2.68)*	57(51-65)	0.55(0.49-0.62)	-5.50(-6.17 to -4.83)*	3599(3225-4076)	34.7(31.09-39.3)	-5.45(-6.13 to -4.78)*
Australasia	9(6-12)	0.43(0.30-0.59)	-1.1(-2.05 to -0.14)*	3(2-4)	0.14(0.11-0.19)	-2.27(-3.01 to -1.53)*	184(141-241)	9.03(6.92-11.81)	-2.25(-2.99 to -1.51)*
Western Europe	127(106-148)	0.48(0.40-0.56)	-2.07(-2.43 to -1.7)*	48(43-54)	0.18(0.16-0.2)	-3.22(-3.64 to -2.8)*	3017(2723-3361)	11.38(10.27-12.67)	-3.18(-3.43 to -2.92)*
Southern Latin America	42(30-57)	0.78(0.57-1.07)	-0.95(-1.50 to -0.4)*	24(19-30)	0.45(0.36-0.56)	-1.60(-2.00 to -1.20)*	1481(1172-1854)	27.87(22.05-34.9)	-1.59(-1.99 to -1.19)*
High-income North America	127(109-149)	0.50(0.43-0.59)	0.12(-0.67 to 0.92)	47(43-52)	0.19(0.17-0.2)	-0.68(-1.25 to -0.12)*	2942(2701-3251)	11.62(10.67-12.84)	-0.67(-1.23 to -0.1)*
Caribbean	31(23-39)	0.83(0.62-1.05)	-0.42(-0.61 to -0.23)*	22(16-29)	0.59(0.43-0.76)	-0.51(-0.68 to -0.34)*	1366(1010-1770)	36.4(26.9-47.16)	-0.51(-0.68 to -0.34)*
Andean Latin America	108(78-144)	2.05(1.48-2.72)	-0.89(-1.48 to -0.3)*	72(53-95)	1.36(1-1.8)	-1.33(-1.88 to -0.78)*	4476(3299-5907)	84.56(62.34-111.61)	-1.33(-1.87 to -0.78)*
Central Latin America	313(257-374)	1.52(1.25-1.82)	0.12(-0.12 to 0.37)	181(150-218)	0.88(0.73-1.06)	-0.55(-0.96 to -0.13)*	11278(9307-13541)	54.96(45.35-65.98)	-0.54(-0.95 to -0.13)*
Tropical Latin America	141(128-155)	0.79(0.72-0.87)	-1.05(-1.30 to -0.80)*	93(84-102)	0.52(0.47-0.58)	-1.40(-1.72 to -1.07)*	5775(5229-6361)	32.51(29.43-35.81)	-1.39(-1.72 to -1.06)*
North Africa and Middle East	419(341-521)	0.80(0.65-1.00)	-1.31(-1.56 to -1.06)*	280(221-353)	0.54(0.43-0.68)	-1.70(-1.96 to -1.43)*	17380(13773-21912)	33.36(26.44-42.06)	-1.69(-1.96 to -1.42)*
South Asia	1318(1113-1534)	0.86(0.73-1.00)	-1.06(-1.87 to -0.25)*	986(836-1147)	0.64(0.55-0.75)	-1.17(-1.97 to -0.36)*	61120(51882-71224)	39.89(33.86-46.48)	-1.17(-1.97 to -0.36)*
Central Sub-Saharan Africa	51(37-69)	0.50(0.36-0.68)	-2.07(-2.15 to -1.99)*	39(28-52)	0.38(0.27-0.51)	-2.04(-2.11 to -1.96)*	2390(1708-3214)	23.56(16.84-31.67)	-2.04(-2.12 to -1.96)*
Eastern Sub-Saharan Africa	173(140-220)	0.54(0.44-0.69)	-2.17(-2.30 to -2.05)*	132(106-171)	0.42(0.33-0.54)	-2.12(-2.23 to -2)*	8200(6573-10620)	25.8(20.68-33.41)	-2.12(-2.23 to -2)*
Southern Sub-Saharan Africa	33(24-43)	0.46(0.33-0.60)	-2.16(-2.88 to -1.43)*	24(18-32)	0.34(0.25-0.45)	-2.23(-2.95 to -1.52)*	1504(1096-1966)	21.18(15.44-27.7)	-2.23(-2.94 to -1.51)*
Western Sub-Saharan Africa	130(102-161)	0.37(0.29-0.47)	-1.37(-1.49 to -1.26)*	99(78-125)	0.29(0.22-0.36)	-1.38(-1.50 to -1.26)*	6138(4807-7726)	17.73(13.89-22.32)	-1.38(-1.50 to -1.26)*

Table S4: Incident cases, deaths, and DALYs of AYA cancers among 30 to 34 years in 2019, and percentage change in age-specific rates from 1990 to 2019, by sex, and cancer types

	Incidence			Deaths			DALYs		
	**Number of incident cases**	**Age-specific incidence rate (per 100 000 population)**	**APC**	**Number of deaths**	**Age-specific death rate (per 100 000 population)**	**APC**	**Number of DALYs**	**Age-specific DALYs rate (per 100 000 population)**	**APC**
Global	15313(13992-16714)	2.54(2.33-2.78)	-0.91(-1.50 to -0.31)*	8296(7639-9004)	1.38(1.27-1.5)	-1.89(-2.21 to -1.58)*	475179(437770-515055)	78.97(72.75-85.6)	-1.88(-2.19 to -1.57)*
Male	8863(7891-9929)	2.92(2.60-3.28)	0.47(-1.00 to 0.05)	4509(4110-4509)	1.49(1.36-1.65)	-1.67(-2.13 to -1.22)*	258669(236197-286332)	85.32(77.91-94.44)	-1.66(-2.11 to -1.20)*
Female	6450(5647-7324)	2.16(1.89-2.45)	-1.37(-2.00 to -0.73)*	3787(3354-4234)	1.27(1.12-1.42)	-2.35(-3.23 to -1.26)*	216510(191643-241943)	72.52(64.19-81.04)	-2.34(-3.22 to -1.45)*
East Asia	7449(6322-8707)	5.61(4.76-6.56)	0.32(-0.63to 1.29)	2879(2462-3339)	2.17(1.86-2.52)	-1.81(-2.91 to -0.70)*	165917(142447-192202)	125(107.32-144.8)	-1.77(-2.87 to -0.66)*
Southeast Asia	659(557-795)	1.25(1.05-1.50)	-2.12(-2.36 to -1.89)*	481(406-562)	0.91(0.77-1.06)	-2.52(-2.70 to -2.35)*	27448(23193-32068)	51.91(43.86-60.64)	-2.52(-2.69 to -2.35)*
Oceania	36(25-50)	3.69(2.55-5.17)	-0.07(-0.22 to 0.08)	29(20-41)	3.01(2.08-4.21)	-0.04(-0.24 to 0.17)	1673(1155-2345)	171.38(118.31-240.15)	-0.04(-0.24 to 0.17)
Central Asia	225(195-259)	2.76(2.39-3.18)	-2.49(-2.98 to -2.00)*	181(157-209)	2.22(1.92-2.56)	-2.61(-3.09 to -2.12)*	10300(8933-11895)	126.28(109.52-145.83)	-2.61(-3.09 to -2.13)*
Central Europe	100(85-117)	1.23(1.05-1.44)	-2.03(-2.23 to -1.83)*	66(56-76)	0.81(0.7-0.94)	-2.66(-2.87 to -2.45)*	3755(3207-4346)	46.49(39.7-53.79)	-2.65(-2.86 to -2.45)*
Eastern Europe	650(563-746)	3.67(3.18-4.21)	-1.17(-1.96 to -0.38)*	342(297-391)	1.93(1.67-2.2)	-2.16(-3.08 to -1.23)*	19600(16994-22314)	110.56(95.86-125.86)	-2.15(-3.06 to -1.23)*
High-income Asia Pacific	460(394-533)	4.26(3.65-4.95)	-3.29(-3.69 to -2.89)*	137(123-152)	1.27(1.14-1.41)	-5.09(-5.49 to -4.69)*	7952(7141-8865)	73.73(66.21-82.19)	-5.05(-5.45 to -4.65)*
Australasia	24(16-33)	1.14(0.79-1.59)	-0.55(-1.35 to 0.25)	8(6-11)	0.4(0.3-0.51)	-1.85(-2.34 to -1.36)*	478(361-618)	23.09(17.46-29.83)	-1.83(-2.31 to -1.33)*
Western Europe	350(298-414)	1.26(1.07-1.49)	-1.61(-1.97 to -1.25)*	138(124-154)	0.5(0.45-0.55)	-2.88(-3.09 to -2.68)*	7955(7175-8858)	28.6(25.8-31.85)	-2.86(-3.06 to -2.66)*
Southern Latin America	86(61-116)	1.70(1.21-2.30)	-1.22(-1.57 to -0.87)*	53(42-65)	1.05(0.83-1.29)	-1.80(-2.30 to -1.29)*	3021(2405-3731)	59.84(47.63-73.89)	-1.79(-2.3 to -1.28)*
High-income North America	283(243-333)	1.15(0.99-1.36)	0.21(-0.37 to 0.79)	111(102-123)	0.45(0.42-0.5)	-0.58(-1.04 to -0.12)*	6399(5869-7090)	26.09(23.93-28.91)	-0.56(-1.02 to -0.11)*
Caribbean	60(45-77)	1.67(1.26-2.16)	-0.5(-0.83 to -0.16)*	46(34-60)	1.3(0.96-1.69)	-0.62(-0.93 to -0.31)*	2642(1961-3444)	73.94(54.89-96.39)	-0.62(-0.92 to -0.31)*
Andean Latin America	183(135-240)	3.81(2.82-5.00)	-0.74(-1.34 to -0.14)*	133(99-173)	2.76(2.05-3.6)	-1.25(-1.82 to -0.67)*	7573(5614-9885)	157.57(116.81-205.67)	-1.24(-1.81 to -0.67)*
Central Latin America	547(452-659)	2.89(2.39-3.48)	-0.10(-0.64 to 0.44)	341(285-410)	1.8(1.51-2.17)	-0.84(-1.18 to -0.5)*	19508(16272-23412)	103.14(86.03-123.78)	-0.83(-1.18 to -0.49)*
Tropical Latin America	304(278-333)	1.68(1.54-1.84)	-1.10(-1.49 to -0.70)*	218(200-238)	1.21(1.11-1.31)	-1.51(-1.92 to -1.11)*	12465(11418-13597)	68.87(63.09-75.13)	-1.51(-1.91 to -1.1)*
North Africa and Middle East	822(683-989)	1.55(1.29-1.87)	-1.42(-1.58 to -1.25)*	588(481-721)	1.11(0.91-1.36)	-1.90(-2.07 to -1.73)*	33574(27443-41094)	63.51(51.91-77.73)	-1.9(-2.07 to -1.72)*
South Asia	2304(1986-2682)	1.63(1.40-1.89)	-1.02(-1.38 to -0.65)*	1899(1622-2215)	1.34(1.14-1.56)	-1.13(-1.51 to -0.75)*	108196(92453-126057)	76.32(65.22-88.92)	-1.13(-1.51 to -0.75)*
Central Sub-Saharan Africa	97(67-134)	1.13(0.78-1.56)	-1.84(-1.88 to -1.8)*	81(56-110)	0.94(0.65-1.28)	-1.80 (-1.85 to -1.76)*	4589(3172-6294)	53.37(36.89-73.21)	-1.81(-1.85 to -1.76)*
Eastern Sub-Saharan Africa	312(252-397)	1.18(0.95-1.50)	-1.94(-2.05 to -1.84)*	262(212-331)	0.99(0.8-1.25)	-1.88(-1.97 to -1.79)*	14949(12063-18817)	56.46(45.56-71.07)	-1.88(-1.97 to -1.79)*
Southern Sub-Saharan Africa	75(56-98)	1.10(0.82-1.43)	-2.13(-3.09 to -1.16)*	61(46-79)	0.89(0.66-1.15)	-2.22(-3.16 to -1.27)*	3479(2596-4497)	50.57(37.74-65.37)	-2.22(-3.16 to -1.26)*
Western Sub-Saharan Africa	287(229-351)	0.99(0.79-1.21)	-1.04(-1.13 to -0.95)*	241(194-295)	0.83(0.67-1.02)	-1.03(-1.14 to -0.93)*	13706(11077-16812)	47.32(38.24-58.04)	-1.03(-1.14 to -0.93)*

Table S5: Incident cases, deaths, and DALYs of AYA cancers among 35 to 39 years in 2019, and percentage change in age-specific rates from 1990 to 2019, by sex, and cancer types

	Incidence			Deaths			DALYs		
	**Number of incident cases**	**Age-specific incidence rate (per 100 000 population)**	**APC**	**Number of deaths**	**Age-specific death rate (per 100 000 population)**	**APC**	**Number of DALYs**	**Age-specific DALYs rate (per 100 000 population)**	**APC**
Global	22296(20443-24399)	4.12(3.78-4.51)	-1.54(-1.84 to -1.24)*	13595(12541-14780)	2.51(2.32-2.73)	-2.35(-2.61 to -2.09)*	709548(653955-770645)	131.16(120.88-142.45)	-2.34(-2.6to -2.07)*
Male	13136(11635-14709)	4.82(4.27-5.40)	-1.22(-1.62 to -0.82)	7569(6828-8368)	2.78(2.51 to 3.07)	-2.16(-2.48 to -1.84)*	395522(356575-436387)	145.12(130.83-160.12)	-2.15(-2.47 to-1.82)*
Female	9160(8202-10174)	3.41(3.06-3.79)	-1.96(-2.10 to -1.82)*	6025(5400-6693)	2.24(2.01-2.49)	-2.57(-2.78 to -2.37)*	314026(281402-348388)	116.98(104.83-129.78)	-2.54(-2.77 to -2.30)*
East Asia	9605(8087-11226)	9.16(7.72-10.71)	-0.23(-0.58 to 0.12)	4364(3698-5047)	4.16(3.53-4.82)	-2.26(-2.48 to -2.04)*	228772(194294-264236)	218.29(185.39-252.13)	-2.23(-2.45 to -2.01)*
Southeast Asia	996(841-1173)	1.94(1.64-2.29)	-2.05(-2.25 to -1.85)*	784(668-919)	1.53(1.3-1.79)	-2.40(-2.60 to -2.20)*	40775(34737-47825)	79.44(67.68-93.18)	-2.39(-2.59 to -2.19)*
Oceania	50(34-72)	5.88(3.98-8.41)	-0.16(-0.35 to 0.04)*	44(30-63)	5.13(3.47-7.32)	-0.15(-0.36 to 0.07)	2287(1549-3264)	266.56(180.63-380.52)	-0.15(-0.36 to 0.07)
Central Asia	336(292-391)	5.00(4.34-5.82)	-2.43(-2.67 to -2.19)*	286(247-332)	4.25(3.68-4.94)	-2.52(-2.77 to -2.28)*	14854(12862-17283)	220.92(191.3-257.05)	-0.15(-0.36 to 0.08)*
Central Europe	202(171-235)	2.38(2.01-2.77)	-2.31(-2.57 to -2.04)*	147(125-170)	1.73(1.47-2.00)	-2.80(-3.06 to -2.54)*	7641(6524-8851)	89.8(76.68-104.03)	-2.79(-3.06 to -2.53)*
Eastern Europe	1088(949-1233)	6.44(5.62-7.30)	-1.53(-2.64 to -0.39)*	645(558-738)	3.82(3.30-4.37)	-2.14(-3.23 to -1.03)*	33694(29144-38537)	199.49(172.55-228.16)	-2.12(-3.22 to -1.02)*
High-income Asia Pacific	995(853-1162)	7.93(6.80-9.26)	-3.38(-4.02 to -2.74)*	299(270-335)	2.39(2.15-2.67)	-4.79(-5.27 to -4.3)*	15873(14318-17772)	126.47(114.07-141.59)	-4.75(-5.23 to -4.27)*
Australasia	37(26-52)	1.84(1.28-2.60)	-0.65(-0.89 to -0.42)*	15(12-20)	0.77(0.6-0.99)	-1.80(-2.00 to -1.6)*	812(629-1041)	40.41(31.3-51.82)	-1.78(-1.98 to -1.57)*
Western Europe	641(537-761)	2.23(1.87-2.65)	-1.63(-1.84 to -1.41)*	290(263-320)	1.01(0.92-1.11)	-2.73(-2.90 to -2.56)*	15228(13809-16755)	52.98(48.05-58.3)	-2.72(-2.89 to -2.54)*
Southern Latin America	146(103-196)	3.04(2.14-4.09)	-0.91(-1.2 to -0.62)*	100(80-122)	2.09(1.67-2.55)	-1.56(-1.92 to -1.2)*	5211(4182-6387)	108.7(87.23-133.24)	-1.55(-1.91 to -1.19)*
High-income North America	446(377-526)	1.84(1.56-2.17)	-0.10(-0.53 to 0.33)	199(184-217)	0.82(0.76-0.9)	-0.91(-1.36 to -0.45)*	10447(9633-11361)	43.19(39.82-46.97)	-0.89(-1.34 to -0.44)*
Caribbean	85(66-110)	2.70(2.10-3.48)	-0.66(-0.99 to -0.33)*	70(54-91)	2.23(1.71-2.87)	-0.75(-1.00 to -0.50)*	3659(2810-4715)	115.88(88.99-149.32)	-0.75(-1.00 to -0.50)*
Andean Latin America	258(191-339)	5.79(4.30-7.61)	-1.03(-1.84 to -0.2)*	202(148-265)	4.54(3.32-5.95)	-1.34(-2.02 to -0.66)*	10515(7693-13775)	236.28(172.88-309.55)	-1.34(-2.02 to -0.66)*
Central Latin America	841(685-1038)	4.73(3.85-5.84)	-0.08(-0.28 to 0.13)	580(476-710)	3.26(2.68-3.99)	-0.69(-0.95 to -0.44)*	30246(24847-36977)	170.09(139.73-207.94)	-0.69(-0.94 to -0.43)*
Tropical Latin America	512(466-564)	2.84(2.59-3.13)	-1.47(-1.57 to -1.38)*	400(366-436)	2.22(2.03-2.42)	-1.81(-1.91 to -1.71)*	20839(19048-22715)	115.63(105.69-126.04)	-1.81(-1.90 to -1.71)*
North Africa and Middle East	1228(1032-1447)	2.51(2.11-2.95)	-1.77(-1.86 to -1.68)*	948(789-1133)	1.94(1.61-2.31)	-2.19(-2.28 to -2.10)*	49347(41074-58983)	100.75(83.86-120.43)	-2.19(-2.27 to -2.1)*
South Asia	3625(3062-4245)	2.84(2.40-3.32)	-1.02(-1.57 to -0.47)*	3157(2702-3691)	2.47(2.11-2.89)	-1.11(-1.69 to -0.53)*	164087(140715-191660)	128.44(110.14-150.02)	-1.12(-1.70 to -0.53)*
Central Sub-Saharan Africa	147(104-204)	2.09(1.47-2.91)	-1.76(-1.85 to -1.67)*	129(90-180)	1.83(1.29-2.56)	-1.73(-1.82 to -1.63)*	6697(4695-9372)	95.31(66.81-133.37)	-1.73(-1.82 to -1.63)*
Eastern Sub-Saharan Africa	495(403-616)	2.21(1.80-2.75)	-1.98(-2.10 to -1.87)*	439(356-549)	1.96(1.59-2.45)	-1.92(-2.02 to -1.81)*	22846(18523-28501)	102.05(82.74-127.31)	-1.92(-2.03 to -1.81)*
Southern Sub-Saharan Africa	123(93-158)	2.08(1.57-2.68)	-2.13(-2.72 to -1.54)*	105(80-134)	1.78(1.36-2.28)	-2.21(-2.78 to -1.64)*	5467(4165-6999)	92.75(70.67-118.75)	'-1.92(-2.03 to -1.81)*
Western Sub-Saharan Africa	440(352-547)	1.82(1.46-2.26)	-1.11(-1.20 to -1.02)*	390(313-486)	1.61(1.30-2.01)	-1.13(-1.21 to -1.04)*	20253(16284-25255)	83.93(67.49-104.67)	-1.13(-1.212to -1.04)*

Table S6: Incidence, deaths and DALYs of AYA stomach cancer in 1990 and 2019 for both sexes and percentage change of age-standardized rates by country and region

	Incidence			Death			DALY		
	1990	2019	Percentage change	1990	2019	Percentag e change	1990	2019	Percentage change
Global	2.34	1.62	-13.21	1.73	0.92	-46.77	96.99	51.96	-46.43
High-income North America	0.71	0.71	-0.71	0.36	0.29	-19.94	20.26	16.28	-19.65
Canada	0.86	0.81	-5.68	0.39	0.26	-33.62	21.72	14.54	-33.02
Greenland	2.04	1.14	-44.21	1.59	0.81	-49.04	87.61	44.68	-49
USA	0.69	0.7	0.26	0.36	0.29	-18.27	20.09	16.47	-18.03
Australasia	0.88	0.69	-24.87	0.46	0.26	-43.56	25.99	14.65	-43.63
Australia	0.79	0.65	-17.35	0.4	0.24	-39.93	22.3	13.38	-39.98
New Zealand	1.36	0.93	-32.11	0.79	0.4	-49.24	44.66	22.66	-49.27
High-income Asia-Pacific	7.62	2.88	-8.17	3.68	0.84	-77.25	207.82	47.58	-77.11
Brunei	4.79	2.1	-56.14	3.29	1.18	-64.09	186.44	66.7	-64.22
Japan	6.91	2.5	-63.86	2.63	0.72	-72.82	147.45	40.76	-72.36
Singapore	2.05	0.74	-64.00	1.09	0.22	-80.15	61.04	12.48	-79.55
South Korea	9.38	3.9	-58.45	6.15	1.15	-81.3	348.49	65.17	-81.3
Western Europe	1.28	0.79	-29.88	0.77	0.33	-56.76	42.82	18.53	-56.74
Andorra	1.28	1.2	-5.89	0.69	0.48	-31.54	38.87	26.74	-31.21
Austria	1.77	0.57	-67.81	1.06	0.24	-77.58	58.93	13.4	-77.27
Belgium	0.83	0.49	-40.07	0.49	0.21	-57	27.66	11.88	-57.04
Cyprus	0.49	0.69	42.04	0.35	0.3	-13.13	19.15	16.87	-11.9
Denmark	0.96	0.47	-51.46	0.6	0.2	-66.63	33.18	11.22	-66.18
Finland	1.58	0.79	-49.83	0.89	0.31	-65.59	49.82	17.37	-65.13
France	0.79	0.64	-19.3	0.49	0.27	-45.01	27.15	14.96	-44.89
Germany	1.27	0.82	-35.76	0.82	0.38	-53.1	45.62	21.38	-53.13
Greece	1.53	1.06	-30.71	0.86	0.47	-44.74	48.49	26.73	-44.87
Iceland	1.35	0.67	-50.69	0.69	0.25	-63.62	38.68	14.12	-63.51
Ireland	0.87	0.63	-27.47	0.54	0.24	-54.68	29.91	13.67	-54.31
Israel	0.87	0.63	-27.47	0.54	0.24	-54.68	40.18	22.51	-43.98
Italy	1.72	1.05	-38.62	0.98	0.41	-58.14	54.94	23.09	-57.97
Luxembourg	1.28	0.62	-51.95	0.79	0.25	-67.82	44.04	14.33	-67.47
Malta	0.83	0.66	-20.29	0.52	0.29	-43.8	29.44	16.61	-43.58
Netherlands	0.97	0.71	-26.38	0.53	0.27	-48.18	29.33	15.3	-47.82

Norway	1.04	0.49	-52.75	0.59	0.19	-68.07	32.86	10.57	-67.84
Portugal	3.67	1.7	-53.61	2.48	0.74	-70.34	139.44	41.1	-70.52
Spain	1.99	1.08	-45.68	1.07	0.37	-65.13	60.01	20.97	-65.05
Sweden	0.78	0.37	-52.58	0.45	0.16	-65.22	25.41	8.93	-64.85
Switzerland	1.51	0.57	-62.21	0.77	0.21	-73.00	43.33	11.71	-72.98
United Kingdom	0.72	0.6	-17.51	0.42	0.26	-37.92	23.76	14.77	-37.81
Southern Latin America	1.54	1.14	-16.69	1.17	0.73	-37.97	65.37	40.74	-37.67
Argentina	1.33	1.04	-21.8	1.02	0.69	-32.05	57.27	39.13	-31.68
Chile	2.02	1.42	-29.72	1.51	0.81	-46.55	83.91	44.95	-46.44
Uruguay	1.55	1.09	-29.66	1.14	0.7	-38.71	63.58	39.19	-38.36
Eastern Europe	3.69	2.37	-9.73	2.62	1.31	-50.16	146.32	73.15	-50.00
Belarus	5.07	2.44	-51.88	3.67	1.29	-64.87	205.82	72.35	-64.85
Estonia	3.79	2.37	-37.4	2.48	0.92	-63.02	139.11	52.12	-62.53
Latvia	3.53	1.77	-49.74	2.53	0.98	-61.29	141.14	54.86	-61.13
Lithuania	3.42	1.74	-49.2	2.42	1.02	-57.85	134.15	56.89	-57.59
Moldova	3.61	1.47	-59.21	2.78	0.99	-64.32	156.27	55.73	-64.34
Russia	3.31	2.31	-30.11	2.22	1.15	-48.31	123.49	64.14	-48.06
Ukraine	4.62	2.66	-42.54	3.66	1.89	-48.25	205.87	106.82	-48.11
Central Europe	1.61	0.85	-29.25	1.28	0.58	-54.93	71.07	32.10	-54.83
Albania	2.02	1.97	-2.39	1.62	1.36	-15.74	92.52	78.11	-15.57
Bosnia and Herzegovina	1.38	1.03	-25.53	1.12	0.75	-33.41	63.00	42.10	-33.18
Bulgaria	1.95	1.11	-42.93	1.56	0.82	-47.32	63.00	42.10	-33.18
Croatia	2.02	0.89	-56.22	1.34	0.44	-67.27	74.35	24.45	-67.11
Czech Republic	1.4	0.69	-50.56	0.99	0.34	-65.22	54.65	19.23	-64.81
Hungary	1.59	0.57	-64.18	1.24	0.38	-69.15	69.04	21.27	-69.19
Macedonia	2.1	1.21	-42.68	1.72	0.88	-48.66	95.50	49.05	-48.64
Montenegro	1.04	0.68	-34.96	0.78	0.46	-41.01	43.62	25.74	-40.99
Poland	1.51	0.69	-54.11	1.28	0.54	-58.28	71.10	29.58	-58.39
Romania	1.60	1.01	-37.1	1.3	0.72	-44.7	72.48	40.07	-44.71
Serbia	1.62	0.89	-45.18	1.24	0.52	-58.01	68.75	28.92	-57.93
Slovakia	1.66	1.02	-38.55	1.11	0.49	-56.06	61.37	27.35	-55.44
Slovenia	1.86	1.04	-43.94	1.18	0.41	-65.67	66.06	22.91	-65.32
Central Asia	3.82	1.9	-13.13	3.14	1.52	-51.67	176.40	85.29	-51.65
Armenia	3.37	1.25	-62.9	2.76	0.94	-66.02	154.16	52.7	-65.81
Azerbaijan	4.20	1.93	-54.1	3.47	1.52	-56.17	196.18	86.42	-55.95
Georgia	3.31	1.96	-40.63	2.62	1.53	-41.72	147.88	86.22	-41.69
Kazakhstan	4.10	1.4	-65.7	3.36	1.08	-67.8	187.99	60.14	-68.01

Kyrgyzstan	5.16	1.96	-61.98	4.29	1.58	-63.16	239.9	87.69	-63.45
Mongolia	4.88	3.48	-28.82	4.21	2.93	-30.34	230.92	160.24	-30.61
Tajikistan	4.5	3.03	-32.69	3.76	2.5	-33.57	211.78	141.29	-33.29
Turkmenistan	2.74	1.29	-53.02	2.26	1.02	-54.99	127.09	57.7	-54.6
Uzbekistan	3.29	1.82	-44.81	2.71	1.45	-46.59	152.5	81.39	-46.63
Central Latin America	2.00	1.94	-1.43	1.55	1.23	-20.74	87.85	69.75	-20.60
Colombia	2.62	2.45	-6.78	2.03	1.41	-30.35	114.21	79.16	-30.69
Costa Rica	3.78	3.01	-20.4	2.58	1.55	-39.77	143.25	87.62	-38.83
El Salvador	2.31	2.40	3.99	1.85	1.58	-14.71	105	89	-15.24
Guatemala	4.45	4.09	-8.02	3.63	3.02	-16.83	207.75	172.26	-17.08
Honduras	3.02	1.39	-53.89	2.45	1.04	-57.57	139.26	58.48	-58.01
Mexico	1.49	1.5	0.84	1.15	0.96	-17.09	65.35	54.11	-17.2
Nicaragua	1.92	1.2	-37.23	1.5	0.75	-49.94	85.15	42.9	-49.62
Panama	2.18	2	-8.3	1.6	1.14	-28.61	90.11	64.72	-28.17
Venezuela	1.61	1.74	7.96	1.25	1.07	-14.45	69.99	60.66	-13.32
Andean Latin America	3.30	2.51	-7.31	2.7	1.81	-32.82	153.17	102.90	-32.82
Bolivia	4.28	2.39	-44.12	3.56	1.88	-47.15	200.24	105.66	-47.23
Ecuador	3.05	2.64	-13.48	2.52	1.91	-24.31	142.44	108.24	-24.01
Peru	3.14	2.48	-20.9	2.53	1.74	-31.06	144.61	99.52	-31.18
Caribbean	1.28	1.09	-11.4	1.02	0.84	-17.49	57.42	47.50	-17.30
Antigua and Barbuda	1.38	0.86	-38.13	1.07	0.59	-45.2	60.25	33.18	-44.93
The Bahamas	1.63	1.37	-16.19	1.28	1.00	-22.15	71.85	56.06	-21.98
Barbados	1.57	1.1	-29.64	1.19	0.75	-37.12	67.21	42.33	-37.02
Belize	0.99	1.33	33.96	0.82	1.02	24.09	46.79	57.57	23.03
Bermuda	0.82	0.48	-41.16	0.58	0.23	-59.33	32.65	13.3	-59.27
Cuba	0.78	0.46	-40.21	0.57	0.28	-49.85	31.76	15.9	-49.94
Dominica	2.93	2.13	-27.51	2.32	1.63	-29.77	130.97	92.1	-29.67
Dominican Republic	1.18	1.27	7.99	0.97	0.97	-0.28	54.96	54.81	-0.29
Grenada	1.83	0.94	-48.58	1.48	0.71	-52.18	83.21	39.7	-52.28
Guyana	1.44	1.21	-15.76	1.19	0.99	-17.19	67.15	55.37	-17.54
Haiti	2.84	1.66	-41.65	2.38	1.39	-41.71	134.15	77.96	-41.89
Jamaica	0.62	0.81	31.18	0.5	0.59	17.46	28.23	33.32	18.04
Puerto Rico	0.92	0.52	-43.31	0.66	0.29	-55.92	36.66	16.23	-55.74
Saint Lucia	2.06	1.28	-37.81	1.67	0.94	-43.68	92.89	52.75	-43.22
Saint Vincent	1.89	1.17	-38.23	1.51	0.87	-42.07	84.3	49.04	-41.82

and the Grenadines									
Suriname	1.18	0.87	-26.14	0.96	0.69	-28.46	53.67	38.46	-28.34
Trinidad and Tobago	0.91	0.46	-49.67	0.72	0.34	-53.09	40.59	19.12	-52.89
Virgin Islands	1.14	0.63	-44.53	0.91	0.47	-48.63	50.95	25.92	-49.12
Tropical Latin America	1.61	1.12	-18.98	1.3	0.81	-37.81	72.81	45.55	-37.45
Brazil	1.62	1.12	-30.67	1.31	0.81	-37.97	73.34	45.76	-37.61
Paraguay	1.13	0.94	-16.78	0.92	0.69	-24.73	51.53	38.86	-24.59
East Asia	3.55	3.59	0.31	2.73	1.47	-46.3	152.27	82.86	-45.58
China	3.55	3.63	2.37	2.74	1.47	-46.41	152.65	82.92	-45.68
North Korea	4.53	2.70	-40.41	3.47	2.01	-42.18	195	113.55	-41.77
Taiwan (Province of China)	2.74	1.83	-33.05	1.72	0.76	-55.59	97	42.94	-55.73
Southeast Asia	1.46	0.81	-30.67	1.2	0.59	-50.69	67.44	33.37	-50.52
Cambodia	2.10	0.99	-53.14	1.74	0.77	-55.63	98.52	43.85	-55.49
Indonesia	1.24	0.63	-49.57	1.03	0.48	-52.73	58.15	27.61	-52.51
Laos	2.42	0.99	-58.88	2	0.8	-60.04	113.03	45.45	-59.79
Malaysia	0.91	0.66	-28.16	0.74	0.44	-39.9	41.28	24.79	-39.95
Maldives	1.08	0.28	-73.63	0.88	0.17	-80.64	49.26	9.6	-80.51
Mauritius	1.39	0.89	-36.18	1.07	0.59	-44.69	59.74	33.27	-44.3
Myanmar	2.47	0.93	-62.3	2.05	0.73	-64.2	116.36	41.86	-64.02
Philippines	1.25	0.69	-44.46	1.01	0.54	-46.72	57.42	30.66	-46.61
Sri Lanka	0.86	0.51	-40.93	0.68	0.32	-52.82	38.64	18.13	-53.08
Seychelles	1.42	0.9	-36.99	1.15	0.63	-45.46	64.02	35.28	-44.88
Thailand	1.61	1.29	-19.59	1.29	0.82	-36.49	72.53	46.23	-36.26
East Timor	1.32	0.71	-46.4	1.11	0.56	-49.04	62.43	32.12	-48.55
Vietnam	1.70	1.06	-37.63	1.40	0.74	-47.24	77.44	41.17	-46.83
Oceania	2.60	2.60	-0.01	2.10	2.09	-0.49	119.21	119.10	-0.10
American Samoa	3.00	2.9	-3.23	2.35	2.18	-7.06	133.40	124.24	-6.87
Federated States of Micronesia	4.26	3.71	-12.88	3.54	2.93	-17.26	199.20	167.07	-16.13
Fiji	1.39	1.12	-19.01	1.12	0.88	-21.28	63.94	50.4	-21.16
Guam	1.06	1.57	48.08	0.75	1.08	44.31	42.99	61.04	41.97
Kiribati	4.52	4.22	-6.71	3.68	3.49	-5.18	208.67	198.59	-4.83
Marshall Islands	3.62	3.74	3.18	2.96	3.04	2.99	169.44	172.17	1.61
Northern Mariana Islands	3.27	2.18	-33.42	2.33	1.37	-41.4	131.70	77.61	-41.07

Papua New Guinea	2.58	2.48	-3.93	2.08	2.00	-4.01	118.32	114.01	-3.64
Samoa	1.48	1.36	-8.12	1.22	1.07	-12.12	67.56	59.49	-11.95
Solomon Islands	6.40	6.59	2.97	5.28	5.30	0.24	297.69	300.06	0.79
Tonga	2.30	2.26	-1.77	1.86	1.73	-6.72	104.35	98.45	-5.66
Vanuatu	2.93	3.24	10.63	2.43	2.66	9.29	136.99	150.29	9.71
North Africa and Middle East	1.63	1.03	-22.46	1.34	0.74	-44.35	75.13	42.04	-44.05
Afghanistan	5.30	3.94	-25.76	4.48	3.31	-25.96	251.87	185.7	-26.27
Algeria	1.01	0.51	-49.27	0.82	0.37	-55.51	46.01	20.46	-55.53
Bahrain	0.75	0.48	-35.49	0.58	0.30	-48.60	33.48	17.1	-48.94
Egypt	0.66	0.40	-39.47	0.55	0.30	-44.44	30.57	17.05	-44.23
Iran	2.22	1.49	-32.93	1.75	0.98	-43.92	99.93	56.14	-43.82
Iraq	0.88	0.77	-12.70	0.72	0.57	-21.46	40.35	31.8	-21.2
Jordan	0.84	0.61	-26.65	0.68	0.41	-39.68	37.83	23.00	-39.21
Kuwait	0.38	0.25	-35.82	0.27	0.13	-50.59	15.04	7.45	-50.48
Lebanon	1.08	0.90	-16.57	0.85	0.50	-41.05	47.68	28.28	-40.68
Libya	0.82	0.58	-28.74	0.66	0.43	-34.77	36.66	23.91	-34.78
Morocco	0.51	0.32	-37.35	0.43	0.25	-41.27	23.8	13.95	-41.39
Palestine	1.79	1.03	-42.14	1.40	0.74	-47.36	80.32	42.3	-47.34
Oman	0.83	0.45	-45.93	0.66	0.27	-58.42	36.85	15.51	-57.91
Qatar	0.54	0.35	-35.21	0.42	0.19	-54.21	23.92	10.91	-54.41
Saudi Arabia	0.39	0.41	3.91	0.38	0.26	-31.61	21.17	14.51	-31.47
Sudan	2.17	1.67	-23.16	1.82	1.26	-30.81	102.35	70.95	-30.68
Syria	1.03	0.67	-35.09	0.83	0.47	-43.75	47.19	26.77	-43.26
Tunisia	0.68	0.55	-18.75	0.54	0.36	-33.72	30.05	19.92	-33.70
Turkey	3.04	1.37	-54.76	2.49	0.88	-64.6	139.47	49.47	-64.53
United Arab Emirates	0.43	0.35	-19.21	0.35	0.26	-25.3	19.30	14.38	-25.46
Yemen	2.05	1.78	-13.15	1.75	1.48	-15.53	97.2	82.56	-15.07
South Asia	1.59	1.17	-16.73	1.32	0.95	-28.43	75.46	53.71	-28.83
Bangladesh	1.32	0.73	-44.9	1.11	0.58	-47.31	62.85	33.14	-47.27
Bhutan	1.28	0.84	-34.79	1.08	0.66	-38.49	60.46	37.35	-38.23
India	1.72	1.27	-26.31	1.42	1.02	-28.07	81.33	58.15	-28.50
Nepal	1.52	0.99	-35.15	1.27	0.8	-36.69	71.25	45.07	-36.74
Pakistan	0.8	0.87	9.26	0.67	0.73	8.36	37.35	40.51	8.47
Southern sub-Saharan Africa	1.37	0.74	-33.71	1.15	0.6	-47.78	63.63	33.36	-47.58
Botswana	1.00	0.75	-24.5	0.81	0.6	-26.25	45.10	33.23	-26.34

Lesotho	0.72	1.11	53.66	0.59	0.88	48.83	32.94	48.67	47.73
Namibia	0.36	0.27	-26	0.3	0.22	-27.56	16.82	12.17	-27.65
South Africa	1.50	0.55	-63.08	1.26	0.45	-64.19	70.04	25.18	-64.05
Swaziland	0.80	0.84	5.07	0.66	0.69	4.18	36.67	38.06	3.80
Zimbabwe	1.14	1.57	38.15	0.92	1.28	39.39	51.11	71.11	39.13
Western sub-Saharan Africa	0.91	0.65	-30.9	0.76	0.55	-27.88	42.42	30.57	-27.95
Benin	1.27	0.9	-28.86	1.06	0.76	-28.22	59.08	42.45	-28.15
Burkina Faso	1.35	1.07	-21.18	1.13	0.9	-20.84	63.1	49.93	-20.87
Cameroon	1.34	0.99	-26.23	1.12	0.82	-26.43	62.39	45.93	-26.39
Cape Verde	3.39	1.25	-63.03	2.85	0.96	-66.25	158.03	53.49	-66.15
Chad	1.26	1.07	-15.7	1.06	0.89	-15.6	58.96	49.81	-15.51
Côte d'Ivoire	1.46	0.96	-33.69	1.21	0.81	-33.11	67.12	44.93	-33.06
The Gambia	0.41	0.33	-18.35	0.34	0.28	-18.55	18.96	15.43	-18.62
Ghana	1.17	0.65	-44.71	0.97	0.53	-45.34	54.27	29.65	-45.36
Guinea	1.69	1.57	-7.39	1.43	1.33	-6.95	79.69	73.94	-7.21
Guinea-Bissau	2.36	1.55	-34.24	1.92	1.28	-33.19	106.55	71.06	-33.31
Liberia	1.22	0.81	-33.34	1.02	0.68	-32.84	57.1	38.25	-33.00
Mali	2.82	1.88	-33.51	2.39	1.59	-33.6	133.38	88.33	-33.77
Mauritania	1.29	0.58	-55.1	1.08	0.48	-55.67	60.17	26.71	-55.61
Niger	1.34	0.94	-29.39	1.12	0.79	-28.75	62.31	44.36	-28.81
Nigeria	0.31	0.23	-26.72	0.26	0.2	-26.04	14.71	10.85	-26.22
São Tomé and Príncipe	1.12	0.86	-23.31	0.97	0.7	-27.33	53.16	39.3	-26.07
Senegal	1.29	0.85	-34.04	1.07	0.71	-33.9	59.71	39.49	-33.85
Sierra Leone	1.11	0.98	-12.17	0.93	0.82	-12	51.68	45.6	-11.76
Togo	1.36	0.96	-29.6	1.14	0.8	-29.62	63.4	44.58	-29.69
Eastern sub-Saharan Africa	1.43	0.8	-30.82	1.18	0.67	-43.05	65.97	37.52	-43.13
Burundi	1.72	1.01	-41.17	1.39	0.84	-39.49	77.52	46.85	-39.56
Comoros	1.08	0.78	-27.66	0.9	0.65	-27.11	50.00	36.50	-26.99
Djibouti	0.99	0.74	-25.03	0.82	0.62	-24.6	45.77	34.48	-24.66
Eritrea	1.54	1.18	-22.98	1.21	0.99	-18.28	67.49	55.00	-18.50
Ethiopia	2.26	0.73	-67.73	1.88	0.61	-67.52	104.63	33.97	-67.53
Kenya	0.89	0.81	-8.57	0.72	0.72	0.04	40.1	39.83	-0.67
Madagascar	1.6	0.88	-45.11	1.34	0.74	-44.46	75.34	41.48	-44.94
Malawi	0.49	0.29	-40.27	0.41	0.24	-40.16	22.86	13.65	-40.31
Mozambique	0.41	0.43	2.60	0.34	0.35	1.34	19.08	19.31	1.19
Rwanda	1.89	0.79	-58.05	1.54	0.66	-57.38	86.1	36.7	-57.38

Somalia	1.57	1.19	-24.02	1.31	1.03	-21.6	72.88	57.1	-21.65
South Sudan	0.96	0.7	-26.78	0.81	0.6	-25.31	45.13	33.75	-25.22
Tanzania	1.13	0.81	-28.97	0.93	0.67	-27.9	51.97	37.4	-28.03
Uganda	1.01	1.01	0.25	0.83	0.83	0.48	46.31	46.55	0.53
Zambia	1.52	1.02	-32.85	1.25	0.84	-32.61	70.18	47.09	-32.9
Central sub-Saharan Africa	1.31	0.77	-31.32	1.07	0.64	-40.46	60.23	35.76	-40.63
Angola	1.55	0.81	-47.57	1.28	0.67	-47.41	71.67	37.66	-47.45
Central African Republic	1.89	1.34	-29.26	1.44	1.04	-27.68	80.57	58.09	-27.9
Congo	1.73	0.75	-56.61	1.39	0.63	-55.17	77.83	34.88	-55.19
DR Congo	1.17	0.74	-37.12	0.97	0.61	-36.61	54.28	34.26	-36.88
Equatorial Guinea	1.55	0.49	-68.17	1.23	0.4	-67.28	69.05	22.57	-67.31
Gabon	1.30	0.62	-52.22	1.06	0.51	-52.01	58.95	28.31	-51.97
